# Urban groundwater quality in sub-Saharan Africa: current status and implications for water security and public health

**DOI:** 10.1007/s10040-016-1516-6

**Published:** 2017-01-18

**Authors:** D. J. Lapworth, D. C. W. Nkhuwa, J. Okotto-Okotto, S. Pedley, M. E. Stuart, M. N. Tijani, J. Wright

**Affiliations:** 1 British Geological Survey, Maclean Building, Wallingford, OX10 8BB UK; 2 University of Zambia, Great East Road Campus, P.O. Box 32379, Lusaka, Zambia; 3 Victoria Institute for Research on Environment and Development (VIRED) International, Rabuour Environment and Development Centre, Kisumu-Nairobi Road, P.O. Box 6423-40103, Kisumu, Kenya; 4 Robens Centre for Public and Environmental Health, University of Surrey, Guildford, GU2 7XH UK; 5 Department of Geology, University of Ibadan, Ibadan, Oyo State Nigeria; 6 Geography and Environment, University of Southampton, Highfield, Southampton, SO17 1BJ UK

**Keywords:** Groundwater quality, Sub-Saharan Africa, Urban groundwater, Nitrate, Microbiological contamination, Health

## Abstract

Groundwater resources are important sources of drinking water in Africa, and they are hugely important in sustaining urban livelihoods and supporting a diverse range of commercial and agricultural activities. Groundwater has an important role in improving health in sub-Saharan Africa (SSA). An estimated 250 million people (40% of the total) live in urban centres across SSA. SSA has experienced a rapid expansion in urban populations since the 1950s, with increased population densities as well as expanding geographical coverage. Estimates suggest that the urban population in SSA will double between 2000 and 2030. The quality status of shallow urban groundwater resources is often very poor due to inadequate waste management and source protection, and poses a significant health risk to users, while deeper borehole sources often provide an important source of good quality drinking water. Given the growth in future demand from this finite resource, as well as potential changes in future climate in this region, a detailed understanding of both water quantity and quality is required to use this resource sustainably. This paper provides a comprehensive assessment of the water quality status, both microbial and chemical, of urban groundwater in SSA across a range of hydrogeological terrains and different groundwater point types. Lower storage basement terrains, which underlie a significant proportion of urban centres in SSA, are particularly vulnerable to contamination. The relationship between mean nitrate concentration and intrinsic aquifer pollution risk is assessed for urban centres across SSA. Current knowledge gaps are identified and future research needs highlighted.

**Electronic supplementary material:**

The online version of this article (doi:10.1007/s10040-016-1516-6) contains supplementary material, which is available to authorized users.

## Resume

Les ressources en eaux souterraines sont des sources importantes d’eau potable en Afrique, et elles sont d’une énorme importance pour les êtres vivants en milieu urbain et pour le soutien d’une gamme diversifiée d’activités commerciales et agricoles. Les eaux souterraines jouent un rôle important en améliorant la santé en Afrique sub-saharienne (ASS). 250 millions de personnes environ (40% du total) vivent dans des centres urbains à travers l’ASS. L’ASS a connu une expansion rapide de ses populations urbaines depuis les années 1950, avec une augmentation à la fois des densités de population et de leur extension géographique. Les estimations prévoient un doublement de la population urbaine de l’ASS entre 2000 et 2030. L’état qualitatif des ressources en eau souterraine urbaines peu profondes est souvent très pauvre du fait de l’insuffisance de la gestion des déchets et de la protection de la ressource, et pose un risque sanitaire significatif pour les utilisateurs, alors que des ressources plus profondes captées par forage fournissent souvent une source importante d’eau potable de bonne qualité. Etant donné la croissance de la demande future de cette ressource limitée, ainsi que le changement climatique potentiel dans cette région dans le futur, une compréhension détaillée de la quantité et la qualité de cette ressource est exigée pour son utilisation durable. Cet article fournit une évaluation complète de l’état qualitatif, à la fois microbien et chimique, des eaux souterraines urbaines en ASS à travers une gamme de contextes hydrogéologiques et de différents types de point d’eaux souterraines. Les terrains du sous-sol de faible emmagasinement, qui sont situés sous une proportion significative de centres urbains en ASS, sont particulièrement vulnérables à la contamination. Le rapport entre la concentration moyenne en nitrate et le risque intrinsèque de pollution des aquifères est évalué pour les centres urbains de l’ASS. Des lacunes actuelles en matière de connaissances sont identifiées et les besoins de recherches futurs sont mis en évidence.

## Resumen

Los recursos hídricos subterráneos son una fuente importante de agua potable en África y son sumamente importantes para mantener los medios de subsistencia urbanos y para apoyar una amplia gama de actividades comerciales y agrícolas. El agua subterránea tiene un papel importante en la mejora de la salud en el África Subsahariana (SSA). Se calcula que 250 millones de personas (40% del total) viven en centros urbanos en el África Subsahariana. El SSA ha experimentado una rápida expansión en las poblaciones urbanas desde la década de 1950, con el aumento de la densidad de población, así como la ampliación de la cobertura geográfica. Las estimaciones sugieren que la población urbana en África Subsahariana se duplicará entre 2000 y 2030. El estado de calidad del agua subterránea somera es a menudo muy pobre debido a la gestión inadecuada de los desechos y de la protección de las fuentes, representando un riesgo significativo para los usuarios, mientras que los pozos más profundos proporcionan una fuente importante de agua potable de buena calidad. Dado el crecimiento de la demanda futura de este recurso finito, así como los cambios potenciales en el clima futuro en esta región, una comprensión detallada de su cantidad y calidad es necesaria para utilizar este recurso de manera sostenible. Este artículo ofrece una evaluación integral del estado de la calidad del agua, tanto microbiana como química, del agua subterránea urbana en el SSA a lo largo de una serie de terrenos hidrogeológicos y diferentes tipos de puntos de agua subterránea. Los basamentos de bajo almacenamiento, que subyacen a una proporción significativa de los centros urbanos del África Subsahariana, son particularmente vulnerables a la contaminación. Se evalúa la relación entre la concentración media de nitratos y el riesgo intrínseco de contaminación acuífera para los centros urbanos a través de la SSA. Se identifican las deficiencias actuales de conocimientos y se destacan las necesidades de futuras investigaciones.

## 摘要

地下水资源是非洲重要的饮用水源,在维系城市生计和支撑各种商业和农业活动中至关重要。地下水在改善撒哈拉以南非洲民众健康方面发挥着重要作用。在撒哈拉以南非洲,预计有2.5亿人居住在城市地区(占总数的40 %)。自从上世纪50年代,本区域城市人口快速膨胀,密度不断增加,面积不断扩大。估测结果显示,撒哈拉以南非洲的城市人口在2000年至2030年间将翻一番。由于废物管理不当及水源污染,城市浅层地下水源水质常常非常差,对用水者产生很大的健康风险,而较深钻井水源常常提供重要的高质量饮水水源。考虑到此有限水源的基础上将来需求的增长以及本地区将来的气候潜在变化,需要详细了解水量和水质状况,以便可持续利用水源。本文从微生物和化学上综合评价了撒哈拉以南非洲各种水质状况地形及不同地下水水点类型的水质状况。在撒哈拉以南非洲,水存储量低的基岩地形在城市中心占有很大的比例,特别容易受到污染。对整个撒哈拉以南非洲的城市中心平均硝酸盐含量和固有的含水层污染风险之间的关系进行了评价。明确了目前的认识空白,并强调了将来的研究需求。

## Resumo

As águas subterrâneas são fontes importantes de água para consumo na África, e são amplamente importantes para sustentar o modo de vida urbano e dar suporte a uma amplitude diversa de atividades comerciais e agrícolas. As águas subterrâneas têm um papel importante no desenvolvimento da saúde na África subsaariana (ASS). São estimadas 250 milhões de pessoas (40% do total) vivendo nos centros urbanos na ASS. ASS tem vivenciado uma rápida expansão na população urbana desde os anos 50, com aumento das densidades populacionais assim como expansão da cobertura geográfica. As estimativas sugerem que a população urbana na ASS dobrará entre 2000 e 2030. As condições de qualidade dos aquíferos livres em áreas urbanas estão frequentemente muito pobres por causa do gerenciamento de perdas e proteção das fontes inadequados, e apresenta um risco significativo à saúde dos usuários, enquanto poços mais profundos frequentemente fornecem fontes importantes de água potável de boa qualidade. Dado o crescimento na demanda futura desse recurso finito, assim como mudanças potenciais no clima futuro da região, um entendimento detalhado da sua quantidade e qualidade é necessário para o uso sustentável deste recurso. Esse estudo fornece uma avaliação compreensiva das condições de qualidade da água, tanto microbiológica quanto química, das águas subterrâneas urbanas na ASS por uma gama de terrenos hidrogeológicos e diferentes tipos pontuais de águas subterrâneas. Terrenos de menor armazenamento de base, que fundamentam uma porção significante dos centros urbanos da ASS, são particularmente vulneráveis a contaminação. A relação entre a concentração média de nitrato e o risco intrínseco de poluição do aquífero é avaliado para os centros urbanos na ASS. Lacunas do conhecimento atual foram identificadas e destaca-se a necessidade de pesquisas futuras.

## Introduction

Groundwater is the largest and most important water resource in Africa (MacDonald et al. 2012). It is often more reliable, in closer proximity to users, less vulnerable to pollution, and more resilient to climate variability than surface water (MacDonald et al. 2011; Lapworth et al. 2013). Access to safe and reliable water is critical for improving health and livelihoods for low-income communities in Africa and elsewhere globally (Hunter et al. 2010). Many expanding urban areas in sub-Saharan Africa (SSA) are dependent on groundwater for at least some, and many cases the majority, of domestic water supply (Adelana et al. 2008; Foster et al. 1999). Over the last three decades there has been a concerted effort to develop improved water supply and sanitation across Africa (Bartram and Cairncross 2010), a recent example being the UN Millennium Development Goals which had the target of halving by 2015 the population without sustainable access to improved sanitation (JMP 2008). Groundwater is considered the centrepiece of improved drinking-water provision in many parts of Africa (Foster et al. 2006). Within urban and peri-urban contexts, this has led to widespread development of groundwater resources beneath and in close proximity to urban centres across SSA. In addition, urban sanitation provision and waste management systems across SSA are inadequate, with an estimated average of 40% coverage for improved sanitation facilities (World Bank 2012). Water treatment options are often very limited and in many cases municipal facilities for waste and water treatment are overloaded or experiencing reduced functionality partly due to limited funding and poor governance. The high population densities found in urban areas has led to the proliferation of unimproved sanitation provision largely through the use of pit latrines, which are often little more than a hole in the ground, and are in very close proximity to wells and springs that are important for domestic use (Stenström 1996).

Across large parts of SSA there is continued and accelerated expansion of urban and peri-urban settlements. A recent study (UNPF 2007) estimates that between 2000 and 2030 Africa’s urban population, compared to rural population, will double and become the majority. Overall, 37% of Africa’s population are currently urbanised and the urban proportion is growing (World Bank 2016a, b).

While groundwater is considered the most resilient source of drinking water across much of Africa, the lack of adequate management of household and industrial waste in many expanding urban centres is a growing concern (Wang et al. 2012). This poor waste management practice has led to the groundwater resources below many urban and peri-urban areas being put under considerable pressure from pollution loading with clear implications for groundwater quality and public health. This is compounded when current and future climate extremes and increased urbanisation are also considered, which may lead to increased flood risks and related disease outbreaks (McMichael et al. 2006; Howard et al. 2016). Per-capita usage is also predicted to rise in line with prosperity which will put additional stress on available groundwater resources, many of which are found on low yielding basement aquifers (MacDonald et al. 2012). Future trajectories of growth of urban centres in SSA, notable for regions in West and East Africa, and increased demands on groundwater resources mean that urban centres are likely to be more dependent on groundwater in the future (UNEP 2008). Together, these factors challenge the security of groundwater resources in many urban areas across SSA.

Groundwater is the critical resource for human survival and economic development in extensive drought-prone areas of south-eastern, eastern and western Africa, especially where the average rainfall is less than 1,000 mm/annum (Foster et al. 2006) and inter-annual rainfall variability is high and there is evidence that this is likely to increase in the future in many regions (Carter and Parker 2009). There are also a number of trans-boundary aquifers which require strategic management in terms of groundwater resources and water quality (Ashton and Turton 2009). The proximity of groundwater sources, and the related reduced infrastructure costs, makes groundwater an ideal resource to target for urban water-resource development (Foster et al. 1998; Taylor and Barrett 1999); however, in SSA the issue of the vulnerability of critical urban groundwater sources to anthropogenic contamination has to date received little attention compared to other regions globally. There is therefore a limited evidence base regarding groundwater quality status in urban centres across SSA with which to inform long-term policy on the development and management of urban groundwater resources.

**Fig. 1:**
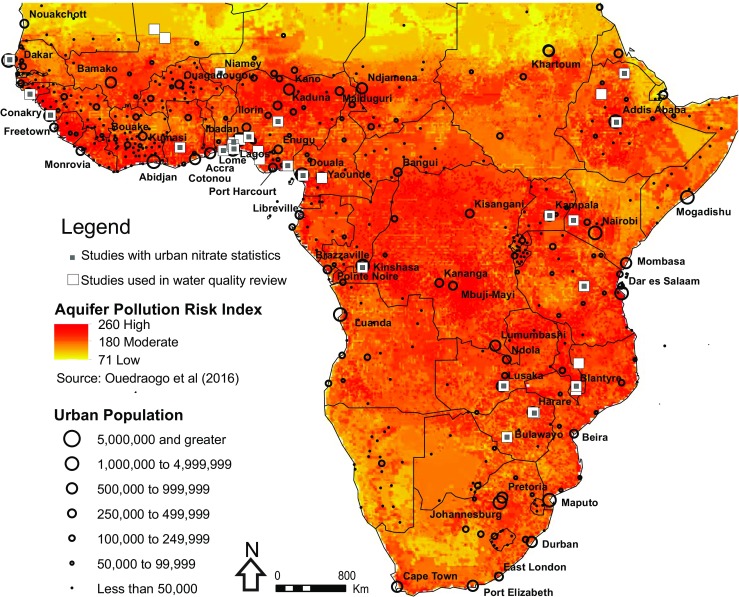
Relationship between urban centres in sub-Saharan Africa (SSA) and estimated aquifer pollution risk using an intrinsic aquifer modelling approach (Ouedraogo et al. 2016). The location of studies included in the paper are shown. Major cities in SSA are shown and are from the ESRI cities dataset (2006)

While regional-scale assessments of groundwater resources have been recently quantified (MacDonald et al. 2012), hydrogeological evidence at the appropriate resolution for managing groundwater resources in many African urban centres are often non-existent. Figure 1 shows the distribution of major urban centres and their relationship to intrinsic aquifer pollution risk using an aquifer vulnerability modelling approach (Ouedraogo et al. 2016). It is noteworthy that the vast majority of urban centres, and in particular urban growth hot-spots in the Lake Victoria Basin and much of West Africa are underlain by high vulnerability aquifers, and are often of moderate-low productivity (MacDonald et al. 2012). It is also clear that the majority of urban centres are located in coastal regions, and as such may be impacted by saline intrusion (Steyl and Dennis 2010), which could be compounded by changes in sea level or over-abstraction of fragile coastal aquifer systems (Comte et al. 2016). Many regions in SSA lack appropriate approaches and adequate investment for groundwater resource planning and management (Braune and Xu 2010). There is also a concern over the decline of many national institutions responsible for groundwater development, resource administration, groundwater protection, as well as loss of professional personnel in the region since the 1980s (Foster et al. 2006), which stems from challenging economic conditions facing SSA as well as poor governance arrangements. An additional concern is the fact that legal frameworks for groundwater resources in SSA do not often cater for community-based arrangements, but rather focus on centralised government permits at the river basin scale.

This paper comprises two parts: firstly, it synthesises existing studies relevant to water quality and health in urban and peri-urban centres and provides an assessment of the current state of knowledge of urban groundwater quality in SSA. This includes an overview of the key issues related to groundwater vulnerability due to anthropogenic contamination, and reviews evidence available from studies assessing key sanitary and non-sanitary sources. The second much smaller part of this paper investigates relationship between estimated aquifer pollution risks, modelled using an intrinsic groundwater vulnerability approach (Aller et al. 1987; Ouedraogo et al. 2016), and observed urban groundwater nitrate concentrations. Nitrate was chosen because it is globally a widely used proxy for anthropogenic contamination in groundwater sources and had suitable data availability across SSA. Although the evidence surrounding the disease burden associated with nitrate in drinking water is complex, high nitrate is frequently associated with high faecal indicator bacteria counts and faecal contamination of urban groundwater in Africa—see Table S1 of the electronic supplementary material (ESM)—which is a known contributor to diarrhoeal disease burden (Prüss-Ustün et al. 2014). Given that more studies included in this review measured nitrate than measured faecal indicator bacteria counts and that nitrate levels are typically less temporally variable than faecal indicator bacteria counts, nitrate levels were examined in this analysis. Empirical studies assessing groundwater quality degradation in urban groundwater in SSA are reviewed, gaps in the current evidence base identified and recommendations made for future research.

## Background: sources and pathways for urban groundwater contamination in sub-Saharan Africa

Microbiological contamination of groundwater and water-related diseases are an important water quality and health challenges for SSA where the health burden disproportionately affects low-income groups and children (Howard et al. 2006). Groundwater contamination can occur whenever there is a source releasing contaminants to the environment (Sililo et al. 2001), a pathway for subsurface transport and a groundwater receptor (see Table 1 for a summary). Faecal waste is the largest source of contamination in urban (and rural) groundwater, in particular where there is high-density housing with poor and/or inadequate sanitation facilities and treatment of faecal waste. This situation is common for low-income areas of most major and growing urban centres in Africa. Across much of SSA, these communities use highly vulnerable shallow sources of groundwater which often provide the majority of drinking water used, in many cases up to 70–80% (Pedley and Howard 1997). Major sources of urban groundwater contamination in SSA include:

**Table 1:** Sources and pathways for urban groundwater contamination in SSA

Component	Category	Risk factors
Regional considerations	–	Population density
		Land use and land cover
		Physical relief/slope
		Rainfall amount and intensity
Sources	Municipal and household level sources including domestic livestock and urban agriculture	*Surface sources:* Open defecation from humans and animals Surface waste sites and incineration sites Fertilisers and pesticides and waste use (solid/Liquid) Atmospheric deposition of combustion products
		*Sub-surface sources:* Pit latrines Septic tanks Soak-aways Waste pits Cemetery or other burial sites Open sewers/drains—most common type in SSA Reticulated sewers—very limit coverage *Other potential sources:* Market places, abattoir waste, both liquid and solid
	Hospital or treatment centre	*Surface and subsurface sources:* Liquid waste discharge to soak-aways/surface channels Solid medical waste disposal Latrines/septic tanks on site
	Industry e.g. mining	*Surface and subsurface sources:* Process plant effluent Solid waste disposal sites Storage tanks including petroleum products Site runoff and leaching from mine spoil
Pathways	Horizontal and vertical pathways in unsaturated and saturated zone	*Shallow sub horizontal pathways in tropical soil*: Tropical soils, e.g. Plithosol/Ferrasol horizons present Shallow depth to water table Thin soils and low organic matter content Natural rapid bypass from tree roots and burrows *Vertical and horizontal pathways in saturated zone:* Thin low-permeability zone above weathered basement Thickness and maturity of weathered basement zone Fracture size, length and density in the more competent bedrock below weathered basement
	Local/headwork pathways	Lack of dugwell headwall and/or lining Lack of well cover Use of bucket and rope—soil/animal/human contact Gap between apron and well lining Damaged well apron Propensity for surface flooding Gap between borehole riser/apron Damaged borehole apron Eroded or de-vegetated spring backfill

- Municipal/domestic waste: for example pit latrines, septic tanks, sewer leakage, sewage effluent, sewage sludge, urban road runoff, landfill/waste dumps and health care facilities
- Industrial sources and waste: for example process waters, plant effluent, stored hydrocarbons, tank and pipeline leakage
- Urban agriculture: for example leached salts, fertilisers, pesticides and animal/human waste
- Mining activities: including both current and historical solid and liquid waste

**Fig. 2:**
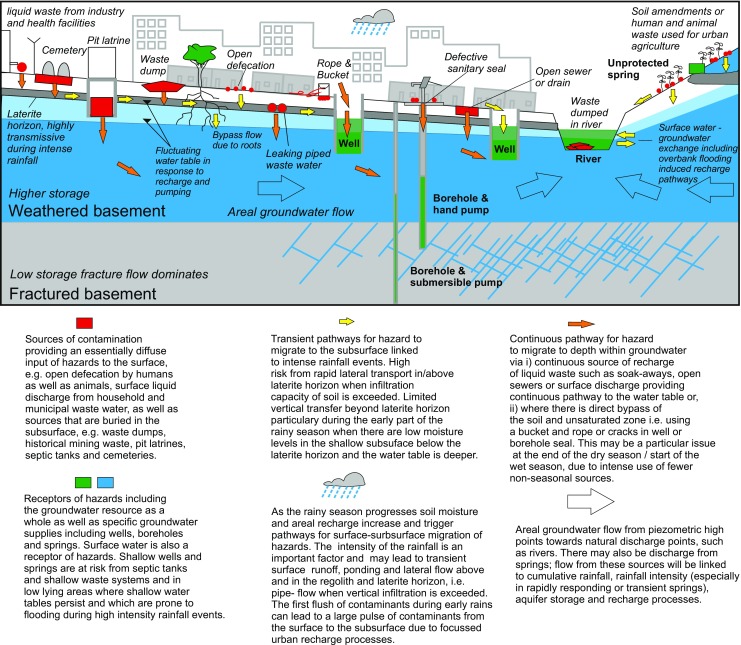
Key potential sources, pathways and receptors of faecal contamination in urban settings in SSA

Figure 2 shows a simplified representation of sources, pathways and receptors for faecal as well as other contaminants in urban SSA. For microorganisms in faecal and other waste materials, the main barrier to their movement into groundwater is the soil and the unsaturated zone. Once into groundwater, there is still a complex interaction of other physical, chemical and biological factors that control the survival and mobility of the microorganisms (Pedley et al. 2006) and influence the distance that they can travel from the source (Stenström 1996; see Table S3 of the ESM).

The causes of water quality contamination may be separated into those related to the production of contaminants at the source and those which govern their delivery into the water environment (Pegram et al. 1999). In peri-urban environments they are primarily caused by infrastructure and services (this includes municipal waste management and treatment and water supply as well as private water and waste services) which are not adequate for the settlement characteristics, are poorly managed by the provider and/or community.

Figure 2 shows a schematic for vulnerable basement (hard rock) settings which are encountered across much of SSA. Most sources and pathways are also relevant for sedimentary settings. As well as moving through the body of the aquifer, contamination can occur via pathways resulting from the design and construction of the supply or its deterioration with time (Fig. 2). Localised contamination is a very common cause of the decline in quality of groundwater supplies, and is frequently illustrated by the rapid rise in the concentration of contaminants following rainfall events (Barrett et al. 2000; Sorensen et al. 2015a). It can occur either where contaminated water:

- Is in direct contact with the headworks of boreholes, wells and springs and where pathways exist that allow this to mix with the water supplies
- Has infiltrated into the sub-surface in the close vicinity of a borehole, well or spring moves along fast horizontal pathways to the supply

Figure 2 summarises key pathways, shown by orange arrows, in high-risk settings such as fractured basement terrains with lateritic soil, including surface and subsurface pathways for migration of pollutants from sources to receptors areas. Very rapid horizontal pathways exist in the shallow tropical soil zone, which may be laterally extensive, in some cases providing transmissivities in excess of 300 m^2^/day (Bonsor et al. 2014). Rapid vertical pathways also exist due to the presence of natural macropores, e.g. from burrows and tree roots as well as soak-aways and other man-made conduits, which can reach significant depths. Combined, these more rapid pathways make shallow wells and spring sources particularly vulnerable to contamination and risks are increased during high groundwater level conditions in response to intense rainfall and recharge and when soil infiltration capacity is exceeded. Horizontal saturated groundwater flow, both in the lower permeability horizon above the weathered basement and in the weathered basement and fractured basement, is a pathway which can affect deeper groundwater sources such as boreholes. These pathways are slower and longer and provide the greatest attenuation potential for hazards.

Surface pathways include surface runoff which can contaminate surface waters and hydrologically connected shallow groundwater sources, as well as poorly protected shallow wells through direct inflow; in addition, there are bypass pathways for contamination of wells and springs via ropes and buckets used to draw water. Shallow sub-surface pathways include vertical soil flow from surface and subsurface sources where there is hydraulic continuity, e.g. from a liquid discharge or from a buried source such as a pit latrine, cemetery or buried waste.

## Data and methods

### Literature review of groundwater quality in urban SSA

There is no systematic monitoring of groundwater quality across Africa, or nationally in most countries, and there are large regions where little or no data are currently available. This section is a compilation of urban groundwater quality data across SSA from a range of literature sources mainly from journal papers and technical reports. Table 2 outlines the literature review primary terms, term descriptions and examples of search terms used for identifying literature. A number of Boolean literature searches were carried out using a range of search engines, Web of Science™, PubMed™, and Google Scholar™. Literature from the searches and other available grey literature was then compiled in an Endnote database (version x7) which included over 500 references after duplicate literature had been removed. The literature was first screened using the article abstract and then subsequently screened using the full article to develop the key water-quality parameters to include in the database. Exclusion criteria included: non-English literature, studies focussed on non-groundwater bodies, studies not undertaken in urban African locations. Key water-quality results were then extracted, summarised and tabulated in a database, and these results are found in Table S1 of the ESM. Quantitative data were obtained for parameters including microbiological indicators, nitrate, and specific electrical conductance (SEC) as well as other trace elements where these are available. Other important meta-data were compiled, for example the geology/soil characteristics, the urban centre, the study type and type groundwater sources included. The final database (*n* = 48) draws mostly on research articles but also includes some results from grey literature including reports and book chapters. These were further subdivided into studies that focussed on assessing risks from pit latrines (*n* = 19) and studies that cover non-sanitary sources of contamination (*n* = 8). It is recognised that these have been published for a range of purposes and with this in mind, the studies can be categorised into three broad groups. These are identified in Table S1 of the ESM as: (1) case-studies (*n* = 22) presenting data from a limited number of sites (*n* < 20), limited temporal resolution as a single survey or use only basic chemical indicators and limited analysis of the results; (2) case studies (*n* = 18) which either draw from larger data sets or include both chemical and microbiological indicators but have limited data analysis regarding sanitary risk factors; and (3) case studies (*n* = 8) with greater temporal resolution or are accompanied by a more thorough analysis of the data, for example using statistical techniques to understand the significance of different risk factors on water quality observations.

**Table 2:** Review terms, term descriptions and search terms used for literature searches and database development

Primary term	Term description	Selected search terms
Population		
Groundwater	Environment, source/pathway of interest	Groundwater; aquifer; well; borehole, spring
Location/landuse	Geographical area of interest	Africa; individual countries; cities; towns
Outcome		
Chemical/physical contamination	Parameters of interest	Contamination; pollution; water quality; nitrate
Microbiological contamination	Organisms or indicator organisms of interest	Faecal/faecal contaminant/coliform; thermotolerant coliforms (TTC); microbe; pathogen; enteric;* E. coli*; virus

### Quantitative assessment of intrinsic aquifer vulnerability index

A subset of studies (*n* = 31) was used for further quantitative assessments for studies that reported suitable nitrate statistics, which was the most common water-quality parameter and a good indicator/tracer of anthropogenic contamination in urban settings. Only studies that fulfilled the following criteria were included: urban study, number of samples reported, mean and standard deviation values reported or could be calculated from raw data. Other important water quality parameters, e.g. thermotolerant coliforms, did not meet the inclusion criteria and had uncertain statistical distributions so were not suitable to carry out similar analysis. Settlement names where these studies were conducted were geocoded using the Google Maps Application Programming Interface (API) version 2. Latitudes and longitudes were returned at the town or city level (accuracy code 4). These coordinates were then linked to a database of urban extents in 2002–2003, derived from Modis satellite data at 1-km resolution (Schneider et al. 2003; Natural Earth 2016), representing each studied settlement’s extent. Among the included studies, 11 studied urban settlements were too small to be mapped in the Modis-derived data, so were represented as points.

The relationship between mean nitrate concentrations and the proportion of boreholes sampled per study, groundwater vulnerability as measured via a DRASTIC risk score (Ouedraogo et al. 2016), and 2010 population density per 1-km^2^ pixel (World Population 2016) was examined. DRASTIC uses a number of parameters including depth to groundwater (D), net recharge (R), aquifer media (A), soil media (S), topography (T), vadose zone (I) and the hydraulic conductivity of the aquifer (C) to develop an index of aquifer vulnerability, see Ouedraogo et al. (2016) for more details on the methods used to develop the aquifer risk score. For each study site’s geographic extent, the mean DRASTIC risk score was extracted as well as the maximum population density per 1-km^2^ pixel. For smaller settlements represented as points, the maximum population density within a 5 km radius was recorded. The latter was chosen as an indicator of the presence of slums with associated high population density and thereby the potential entry of untreated human waste into groundwater and associated nitrate load. The interaction of pollutant loading and aquifer vulnerability should affect observed nitrate levels, rather than these variables operating independently of one another; therefore, the mean nitrate at each site was plotted as a bubble plot against population density (an indicator of nitrate load) and DRASTIC score (an indicator of groundwater vulnerability). Separate plots were produced for boreholes versus wells and springs, since for boreholes, the greater vertical separation between hazards such as septic tanks and the aquifer should reduce observed nitrate (e.g. Sorensen et al. 2015a, b). To examine these relationships statistically, the standard error of mean nitrate per study was calculated, as the standard deviation of nitrate divided by the square of the number of samples, assuming simple random sampling in each study. Random effects meta-regression (Harbord and Higgins 2008) in Stata version 13 was then used to estimate the relationship between reported mean nitrate per study, DRASTIC risk score, and population density. There was no adjustment for clustering in nitrate levels measured at the same city/town in different studies, since such clustering was not statistically significant (individual intra-class correlation coefficient = 0.44 for 7 such city/towns, 95% confidence intervals −0.33 to 0.87).

## Results and discussion

The following sections outline the key findings from the literature review, focussing on anthropogenic contamination of groundwater in urban SSA. While it is beyond the scope of this paper, geogenic sources of contamination are also a major water quality and health issue for some regions in SSA, which includes high probability of elevated As and F above WHO limits (WHO 2011) of 10 ppb and 1.5 mg/L respectively across parts of SSA due to naturally occurring geological sources of contamination. High Fe and Mn are associated with basement aquifers and there is considerable debate as to whether this is naturally occurring or due to corrosion of infrastructure by chemically aggressive groundwater (e.g. Casey et al. 2016). High F groundwater is commonly found associated with volcanic deposits (e.g. Olaka et al. 2016); high As groundwater is commonly associated with sedimentary aquifers as well as mineralised basement settings, and elevated concentrations have been found in mining areas (Smedley 1996). Naturally occurring high salinity/brackish groundwater sources are also found within some arid and coastal settings (e.g. Tole 1997) and the latter may be impacted by over-pumping and/or changes in sea level (Comte et al. 2016)

### Physical, chemical and microbiological indicators of groundwater contamination

It is clear from looking across the published literature (Table S1 of the ESM) that there has been a large number of groundwater quality related studies in southern Nigeria which account for around 30% of the published studies overall (Table S1 of the ESM) and most fall into either category 1 or 2 studies. Most of these studies are located near Lagos, Abeokuta and Ibadan in the south west, the Delta area in the south and Calabar. Other notable examples of locations that have a larger number of case studies include Lusaka in Zambia, Kampala in Uganda, Dakar in Senegal, and Addis Ababa in Ethiopia. Kampala (basement setting), Addis Ababa and Lusaka (basement and karstic settings) all have vulnerable hydrogeological settings (Fig. 1).This section assesses physical and chemical indicators of groundwater contamination available from urban water quality studies across SSA in a range of hydrogeological settings. Studies from group 3 provide the greatest insights regarding pollution sources, pathways and risk factors and can be considered as benchmark studies.

#### Turbidity, total dissolved solids and specific electrical conductance

Turbidity and total dissolved solids (TDS) or specific electrical conductance (SEC) are the most commonly applied physical and chemical water quality indicators used in groundwater studies and are often used in combination with more specific indicators such as nitrate or microbiology (see Table 3). These parameters have a major advantage of being field methods, which are easy to collect enabling surveyors to carry out a simple assessment of water quality rapidly and with minimal cost. For example, high SEC of >1,000–2,000 μS/cm for shallow dug wells (<10 m) of Ibadan metropolis in Nigeria were attributed to contamination from household septic tanks and pit latrines compared to low SEC of <500 μS/cm for deeper wells and boreholes (Tijani and Onodera 2005). The good natural quality of shallow groundwater, with generally relatively low turbidity and low TDS, makes turbidity and TDS good indicators of anthropogenic contamination. Many studies also use hardness or field alkalinity in the same manner, as these are also indicative of contaminant loading and have many of the benefits outlined above (Table S1 of the ESM).

**Table 3:** Studies investigating groundwater contamination from pit latrines in urban SSA (and selected rural settings for comparison) after Lapworth et al. (2015)

Region/country (rural/urban)	Geology/subsurface conditions	Sample sites (*n*)	Water quality parameters	Sampling time frame	Conclusion	Reference
Kulanda town in Bo, Sierra Leone (urban)	Weathered granitic basement	Wells (33), lined and unlined	FC, SEC, NO_3_, Turb, inorganic majors, pH	Wet season	No statistical significance found for pit latrine distance, lowest* P* value (0.06) for distance from field. Low pH concern for corrosion.	Jimmy et al. (2013)
Kamangira, Zimbabwe (rural)	Sandy soils over fractured basement	Installed test wells (17)	NH_4_, NO_3_, turb, pH, Conductivity, TC, FC	Feb–May 2005	Low FC >5 m from PL, N conc. usually below WHO standards	Dzwairo et al. (2006)
Epworth, Zimbabwe (urban)	Fine sandy soils over fractured basement	New and existing wells (18) and boreholes (10)	Na, Zn, Cu, Fe, PO_4_, NO_2_, TC, FC	N/A	Elevated N and coliforms in most of the study area	Zingoni et al. (2005)
Epworth, Zimbabwe (urban)	Fine sandy soils over fractured basement	Installed wells	N, SO_4_, FC	2-8 week intervals 1998–1999	Rapid reduction in coliforms, S and N 5–20 m from PL	Chidavaenzi et al. (2000)
Lusaka, Zambia (urban)	Thin soils and karstic dolomite	Existing wells (NA)	NO_3_, Cl, FC	November 2003, March 2004, October 2004	Greatest FC loading from PL and other waste sources in wet season and dilution of N pollution	Nkhuwa (2006)
Kabwe, Zambia (urban)	Thick overburden and karstic dolomite	Existing wells and boreholes (75)	TTC, NO_3_, Cl	Wet and dry seasons: Sep 2013 and Jan 2014	Greatest TTC and NO_3_ in shallow wells. Significantly better water quality in boreholes. Higher TTCs in the wet season compared to dry season	Sorensen et al. (2015b)
Dakar, Senegal (urban)	Fine-course sands over sediments	Existing wells (47)	Broad hydrochemistry, FC	July and November 1989	Nitrate strongly linked to PL proximity	Tandia et al. (1999)
NW Province, South Africa (rural)	N/A	Existing wells (9)	NH_4_, NO_3_, NO_2_	June–July	High contamination <11 m from PL	Vinger et al. (2012)
Mbazwana, South Africa (urban)	Sands	Installed test wells (5)	FC and NO_3_	Bimonthly 2000–2002	Low nitrate (<10 mg/L) and FC (<10/100 ml) >1 m from PL	Still and Nash (2002)
Bostwana, Mochudi/Ramotswa (rural)	Well–poorly drained soils	Existing wells (>60)	P, N, stable isotopes and Cl	N/A	Variable N leaching from PL	Lagerstedt et al. (1994)
Botswana (rural)	Fractured basement	Existing well and observation well (2)	Broad hydrochemistry, *E. coli*	October–February 1977	Contamination of wells near latrine with *E. coli* and nitrate	Lewis et al. (1980)
Various, Benin (rural)	N/A	Existing wells (225)	Andenovirus, rotavirus	Wet/dry season 2003–2007	Viral contamination is linked to PL proximity	Verheyen et al. (2009)
Langas, Kenya (urban)	N/A	Existing wells (35)	TC, FC	January–June 1999	97% wells positive for FC, 40% of wells >15 m from PL	Kimani-Murage and Ngindu (2007)
Kisumu, Kenya (urban)	Sedimentary	Existing wells (191)	FC, NO_3_, Cl	1998 to 2004	Density of PL within a 100 m radius was significantly correlated with nitrate and Cl but not FC (PC)	Wright et al. (2013)
South Lunzu, Blantyre, Malawi (urban)	Weathered basement	Borehole, springs and dug well (4)	SEC, Cl, Fe, FC, FS	Wet and dry season on two occasions	Groundwaters highly contaminated due to poor sanitation and domestic waste disposal. 58% of residence use traditional PL	Palamuleni (2002)
Uganda, Kampala (urban)	Weathered basement	Piezometers (10)	NO_3_, Cl, PO_4_	March–August 2010 biweekly sampling	PL found to be a significant source of nutrients (N) compared to waste dump	Nyenje et al. (2013)
Uganda, Kampala (urban)	Weathered basement	Installed wells and spring (17)	SEC, pH, P, NO_3_, Cl, FC and FS	March–August 2003, weekly and monthly	Widespread well contamination linked to PL and other waste sources	Kulabako et al. (2007)
Uganda, Kampala (urban)	Weathered basement	Springs (4)	FC, FS, NO_3_, NH_4_	Wet and dry season for 5 consecutive weeks	Widespread contamination from PL and poor animal husbandry, both protected and unprotected sources unfit for drinking	Nsubuga et al. (2004)
Uganda, Kampala (urban)	Weathered basement	Springs (25)	FC, FS	Monthly September 1998–March 1999	Spring contamination linked to local environmental hygiene and completion rather than on-site sanitation (LR)	Howard et al. (2003)
Lichinga, Mozambique (urban)	Mudstone	Lichinga (25)	TTC, EF (Enterococi)	Monthly for 1 year	Higher risk at onset of the wet season and end of the dry season. Predominant source was from animal faeces rather than PL or septic tanks (LR)	Godfrey et al. (2006)

*PL* pit latrine; *FC* faecal coliform (values given as 0 are below detection limit of method), *SEC* specific electrical conductivity, *TTC* thermotolerant coliforms, *TC* total coliform, *FS* faecal strep, *Turb* turbidity, *LR* logistic regression, *PC* Pearson’s correlation. Concentrations in mg/L unless otherwise stated

#### Nitrate and chloride

Nitrate and chloride are the most widely used water-quality indicators of anthropogenic pollution in the studies conducted in SSA, with nitrate having been used in over 80% of the groundwater studies summarised in Table S1 of the ESM The relatively simple sample preservation and analysis required makes these parameters attractive for initial water-quality screening. Nitrate concentrations ranged from below detection limit (BDL) to >500 mg/L, although typical maximum concentrations were generally below 150 mg/L, however this concentration does exceed the WHO drinking-water guideline value for nitrate of 50 mg/L (as nitrate) by a factor of 3 (Table S1 of the ESM ).

Both tracers have been used in a broad range of geologic and climatic zones to investigate pollution from on-site sanitation and waste dumps, as well as urban agriculture (Table 3). Nitrate concentrations show a high degree of variability both within studies and between studies that have been reviewed. Two principal factors that affect nitrate occurrence are firstly the prevailing redox conditions in groundwater, and secondly the residence time and vulnerability of the groundwater body. There are several examples of low nitrate groundwater (Table 3) which show evidence of faecal contamination (Gélinas et al. 1996; Mwendera et al. 2003; Nkhuwa 2003). This suggests the potential for denitrification in shallow groundwater. Nitrate has been used successfully to characterise urban loading to groundwater from a range of sources including pit latrines (Cissé Faye et al. 2004; Tijani and Onodera 2005) and landfills (Ugbaja and Edet 2004; Vala et al. 2011) and was applied to look at impacts on groundwater quality across different population densities (Goshu and Akoma 2011; Goshu et al. 2010; Orebiyi et al. 2010). There are other sources of N loading to groundwater in growing urban areas including the impact of deforestations, and these have been delineated using N:Cl ratios and in a few examples by using δ^15^N analysis (Faillat 1990).

A series of geochemical transformations can occur in water with a high carbon loading with a progressive decline in redox potential, leading sequentially to the removal of nitrate by denitrification, the mobilisation of manganese and iron and the reduction of sulphate. Borehole mixing processes can cause dilution and overall low nitrate concentrations while still having significant microbiologic contamination. Lagerstedt et al. (1994) and Cronin et al. (2007) have successfully used NO_3_:Cl to fingerprint different sources of urban and peri-urban pollution in groundwaters in SSA; this has a certain appeal due to its simplicity; however, prevailing redox conditions and mixing processes need to be considered when using this approach. Many studies have effectively used nitrate in combination with other basic physical indicators such as SEC or TDS and turbidity to assess contamination and map areas of high and low pollution. For example, in Ibadan metropolis, Nigeria, high-SEC shallow dug wells (<10 mbgl) are associated with very high nitrate contamination (NO_3_ of >50–412 mg/L) through inputs of leachates from in-house soak-aways and pit latrines (Tijani and Onodera 2005).

#### Ammonium and phosphate

It is evident from the literature that only a minority of case studies (ca. 20% of studies in Table S1 of the ESM) contain data for NH_4_ and close to 30% contain data for PO_4_. This is in part due to the more involved analytical procedures for NH_4_, the high detection limits for PO_4_ and the fact that these parameters need to be analysed rapidly to ensure valid results.

Ammonium and phosphate are closely associated with contamination from pit latrines and leaking sewer systems and examples from the cities of Lusaka, Abeokuta, Calabar and Makelle are shown in Table S1 of the ESM (Berhane and Walraevens 2013; Cidu et al. 2003; Taiwo et al. 2011; Ugbaja and Edet 2004). Ammonium concentrations in urban groundwater range from BDL to 60 mg/L, although most case studies had maximum concentrations below 10 mg/L. The highest concentrations were reported in Lusaka, Zambia, where the karstic limestone aquifer which underlies much of the city and very rapid transport times in the groundwater are implicated. Both indicators do not behave conservatively in soils and groundwater. NH_4_ is positively charged and therefore has a strong affinity for negatively charged surfaces such as clays; for this reason, as well as microbiological processing, attenuation is particularly high in the soil zone.

Phosphate concentrations range from BDL to 86 mg/L, although very few studies report values >20 mg/L. Phosphate has very limited mobility in the subsurface and has a strong affinity to iron oxy-hydroxides as well as carbonates. Background concentrations are usually low, e.g. <0.2 mg/L; concentrations in urban groundwater are also usually low unless there is either a very high loading or very rapid groundwater flow for example in fractured basement or karstic limestone (Cidu et al. 2003; Nkansah et al. 2010; Zingoni et al. 2005).

#### Trace elements including heavy metals

Overall, relatively few studies have characterised trace element contamination in urban groundwater, and studies published to date usually report results for only a handful of elements (e.g. Fe, Mn, Pb, Zn, Ni, V, Cr, Cd). This is in part due to the cost and access to suitable analytical facilities in SSA for multi-element (ICP-MS or AES), and the relatively poor detection limits for some single element methods.

Studies investigating trace element concentrations have tended to be focused on non-sanitary sources, either the effect of mining (Ikenaka et al. 2010; Nachiyunde et al. 2013a, b; von der Heyden and New 2004) or waste dumps (Momodu and Anyakora 2010; Yusuf 2007). The main findings from these studies are summarised in Table 4. Low concentrations of Cd (0.13–0.2 μg/L) and Pb (0.04–0.09 μg/L) were reported by Vala et al. (2011) in a study in Kinshasa, DRC, where groundwater was contaminated from waste dumps. Aremu et al. (2002) report high Pb (400 μg/L) and moderate Cr (9 μg/L) and Cd (8.5 μg/L) in groundwater from the Warri River plain in Nigeria, and contamination from industry is cited as the source of contamination. von der Heyden and New (2004) reported elevated concentrations of Co, Ni and Zn down flow gradient of tailings, however concentrations were found to be below WHO drinking-water quality standards in all cases, this is likely due to sulphide precipitation and natural attenuation processes.

**Table 4:** Studies focused on impacts of non-sanitary sources on urban groundwater quality in SSA

Area/country	Geology	Sample sites	Results (mg/L), range and/or mean where available		Sources	Reference
Ojota, Nigeria	Sedimentary	10 boreholes, 10 dug wells	SEC 68–3030, mean 584 μS/cm Fe 0–21.4, mean 4.23 Cu 0–33, mean 0.02 Pb 0–14.8, mean 2.4 Zn 0–0.23, mean 0.04		Industrial areas and landfill, Sites within 2 km radius of landfill affected.	Oyeku and Eludoyin (2010)
Akure, Nigeria	Basement complex	Boreholes in landfill vicinity	TDS 18–342 NO_3_ 30–61 Fe 0.9–1.4 Pb 0–1.21 Zn 0–2.3 Cr 0–0.25		Landfill values decrease with distance 50–100 m	Akinbile and Yusoff (2011)
Igando, Lagos, Nigeria	Sedimentary	Wells 10–375 m from landfill	TDS 3–23, mean 9.0 NO_3_ 17.4–60.5, mean 38.5 NH_4_ 0.12–0.3, mean 0.22 PO4 7.07–15.12, mean 10.7		Municipal landfill	Longe and Balogun (2009)
Ibadan, Nigeria	Basement complex	Soil and groundwater	Cd 0.01 Cr, Pb, Co, Ni not detected		Municipal refuse dumps	Adelekan and Alawode (2011)
Ilorin, Nigeria	Basement complex		Colour, turbidity over WHO limit *E. coli* 161–731 TC 1600–>1,800		Industrial estate	Adekunle (2009)
Lokpaukwu, Lekwesi and Ishiagu, Nigeria	Shales and igneous intrusions	Springs and open dug wells	*Dry season* TDS 25–3,150 Cl 0–30 NO_3_–N 0.04–0.74 SO_4_ 0–33.6 Fe 0–3.98 mg/L Mn 0–0.21 mg/L Pb BDL Zn 0–0.06 mg/L Cd 0–0.258 mg/L	*Wet season* TDS 33–11,126 Cl 2.1–1155 NO_3_–N 0.04–0.68 SO_4_ 1–381 Fe 0–5.07 mg/L Mn 0–0.82 mg/L Pb 0–0.24 mg/L Zn 0–1.07 mg/L Cd 0–0.196 mg/L	Mining	Ezekwe et al. (2012)
Dar-es-Salaam, Tanzania	Sedimentary	Wells up and down gradient	*Dry up gradient* Mn 0.03 Fe 0.07 FC (cfu x 10^4^/100 ml) 1.5 *E. coli *5400 SO_4_ 76 *Dry down gradient* Mn 0.02 Fe 0.12 FC (cfu x 10^4^/100 ml) 3.4 *E. coli* 6500 SO_4_ 49	*Wet up gradient* Mn 0.00 Fe 0.12 FC (cfu x 10^4^/100 ml) 0.7 *E. coli* 5000 SO_4_ 35 *Wet down gradient* Mn 0.05 Fe 0.24 FC (cfu x 10^4^/100 ml) 3.7 *E. coli *5300 SO_4_ 72	Solid waste disposal	Kassenga and Mbuligwe (2009)
Lusaka and Copperbelt, Zambia	Dolomites	Surface and groundwater	As 0–0.506, mean 0.009 Cr 0–0.089, mean 0.01 Cu 0–0.270, mean 0.012 Mn 0–10.4, mean 0.369 Ni 0–0.698, mean 0.015 Pb 0–0.094, mean 0.003 Zn 0–1.21, mean 0.75		Mining: Mn, Cu and Ni correlated	Nachiyunde et al. (2013a)

*FC* faecal coliform (values given as 0 are below detection limit of method), *SEC* specific electrical conductivity, *TC* total coliform, *TDS* total dissolved solids. Concentrations in mg/L unless otherwise stated

There have been three notable studies in Addis Ababa, Ethiopia, that have considered natural geogenic contamination within an urban context (Abiye 2008; Alemayehu 2006; Demlie and Wohnlich 2006). These studies report elevated concentrations of Zn, Ni, Pb, Cd, Co and Cr, all above WHO drinking-water-quality standards.

#### Micro-organic pollutants

Micro-organic pollutants include a diverse range of anthropogenic chemicals including pesticides, industrial compounds, pharmaceuticals as well as by-product of water treatment such as trihalomethane which can be generated as a result of chlorination. There has been very little published research on micro-organic contamination in African groundwater systems (Lapworth et al. 2012) and most of the references in the section below are in addition to those found in the literature search and relate to potential sources of micro-organics in groundwater. This is largely due to the costly and complex analytical methods required to undertake such work. The majority of studies that include some micro-organic analysis have been undertaken in South Africa, and few studies include groundwater samples. In urban areas, groundwater is likely to be impacted by anthropogenic micro-organic contaminants from sewage and industrial activities as well as historical waste management practices. Diffuse leakage from reticulated sewerage systems poses a significant pollution risk as it bypasses natural attenuation mechanisms in the subsurface (Ellis 2006). Wastewater may contain pharmaceuticals, household detergents, fragrances and flavourings and plant and animals steroids. Hospital and medical wastewater forms an important source of contaminants including a wide range of pharmaceuticals (Verlicchi et al. 2010; Watkinson et al. 2009), while industrial compounds include solvents, detergents, flame retardants and polyaromatic hydrocarbons (PAH).

A handful of studies have characterised pesticide contamination in freshwater systems in Africa (e.g. Karlsson et al. 2000; Schulz et al. 2003; Awofolu and Fatoki 2004), and pesticide storage and use remains largely unregulated and monitored (Dalvie et al. 2006; London et al. 2005). There are few studies that have investigated micro-organic compounds such as pharmaceuticals in freshwater sources (Hughes et al. 2012; Oketola and Fagbemigun 2013). Sorensen et al. (2015c) provided the first assessment of groundwater contamination for a broad range of compounds (*n* > 1,000) including pharmaceuticals, personal care products and industrial breakdown products within urban groundwater in Africa. Lin et al. (2013) recently characterised a broad range of volatile organic compounds from pit latrines in Africa, an important source of groundwater contamination in this region (Graham and Polizzotto 2013).

Olujimi et al. (2010) review the presence of phthalates and other endocrine disruption chemicals in the South African environment. Fatoki et al. (2010) studied phthalates in surface water and found concentrations which gave cause for concern with diethylhexyl phthalate (DEHP) posing the greatest risk (range 0.3–2.78 mg/L). Ogunfowokan et al. (2006) assessed phthalates in a sewage lagoon and adjacent surface water. Both were assessed as being polluted by a range of phthalate compounds. These were found present at monthly mean concentrations of between 24 and 139 mg/L in the lagoon and 10 and 80 mg/L in the receiving stream. Adeniyi et al. (2008) looked at concentrations of phthalates in soils surrounding an open solid waste dump in Limpopo Province, South Africa. For the phthalate esters dimethyl phthalate (DMP), diethyl phthalate (DEP), dibutyl phthalate (DBP) and DEHP, the mean values calculated were 0.31 ± 0.12, 0.21 ± 0.05, 0.30 ± 0.07, and 0.03 ± 0.01 mg/kg respectively. Mahomed et al. (2008) estimated oestrogenic activity from phthalates in industrial discharges. As part of a wider study Aneck-Hahn et al. (2009) analysed the oestrogenic activity of borehole and spring samples in two communities in Limpopo Province, one rural and one close to a platinum mine. The majority of samples showed such activity. A recent study by Arinaitwe et al. (2014) showed elevated polybrominated diphenyl ethers and alternative flame retardants in air and rainfall samples in East Africa

#### Pathogens and microbiological indicators

The main source of pathogen contamination in water is faeces. Human and animal faeces contains a high microbial load with a very diverse range of species (Leclerc et al. 2001) that are IMW70355derived from the normal flora of the gut. Many of the bacteria in the gut are either difficult to culture, or cannot be cultured with the techniques currently available; therefore, estimating the bacterial load in faeces is extremely difficult. Using methods that detect genetic material, O’Hara and Shanahan (2006) have estimated between 10^11^ and 10^12^ bacteria in one gram of colonic content (60% of the faecal mass). Most of these bacteria are harmless saprophytes that colonise the gut and aid digestion, but faeces also contains pathogenic microorganisms, and it is these that are of concern when they get into water.

The majority of pathogens are difficult or impossible to culture with currently available methods. Although rapid and sensitive methods are being developed and are under trial, they are yet to be widely adopted by the water industry. Where culture methods are available it can take several days before the presence of the pathogen is confirmed, during which time water consumers will have been exposed to the pathogen. To overcome some of these difficulties, a group of indicator bacteria are used routinely to highlight faecal contamination in water. The pathogens that have been isolated from groundwater, and the indicator organisms that have been used to assess risk are described in detail elsewhere in this special issue of *Hydrogeology Journal*.

### Impacts from in-situ sanitation

In-situ sanitation, largely in the form of pit latrines and septic tanks is considered the dominant cause of microbiological contamination and a major cause of nutrient loading to water sources in SSA. There are however other sources of contamination including open defecation (Nkhuwa 2006; Nyenje et al. 2013) and surface ponds and it is often difficult to resolve multiple sources of faecal contamination in groundwater (Howard et al. 2003). Impacts from pit latrines is a well-studied and documented source of groundwater contamination and a worldwide review has been published recently by Graham and Polizzotto (2013). The main findings from studies carried out in SSA have been collated in Table 3 and are summarised below along with other studies specifically targeting contamination from pit latrines.

#### Microbiological contaminants

Human faeces harbour a large number of microbes, including bacteria, archaea, microbial eukarya, viruses, protozoa, and helminths (Graham and Polizzotto 2013). In the context of this review, there have been no studies that have assessed protozoa or helminths, which exhibit little movement in groundwater due to their size (Lewis et al. 1982). The characteristics of microorganisms and the aquifer and soil environment that affect microbial transport and attenuation in groundwater are shown in Table S2 of the ESM.

Microorganisms have been assumed to be rapidly attenuated after excretion but recent studies with viruses suggest that water quality may be impaired for a considerable length of time. Using a mixture of routine culture methods and genetic detection methods, Charles et al. (2009) detected viruses over 300 days after they were introduced in simulated groundwater systems. A number of approaches have been used to define the quantities and transport distances of latrine-derived microbial contaminants. The majority of these have been culture-based studies of faecal bacteria; there has only been one study of viruses related to pit latrines (Verheyen et al. 2009).

Attenuation of microbes is likely to be dependent on the hydrological conditions both in terms of water levels and recharge rate and permeability of the aquifer, and is highly variable (Taylor et al. 2010). Dzwairo et al. (2006) found faecal and total coliforms greatly reduced beyond 5 m from pit latrines in Zimbabwe, whereas Still and Nash (2002) found faecal coliforms to be attenuated to <10 cfu/100 ml after 1 m in Maputaland, KwaZulu-Natal. In Abeokuta, Nigeria, Sangodoyin (1993) found coliform attenuation to be correlated both with distance from the source and with the depth of the groundwater well. In Epworth, Zimbabwe, groundwater contamination was higher in the dry season rather than in the wet, with coliforms detected up to 20 m from the pit (Chidavaenzi et al. 1997). In Benin, Verheyen et al. (2009) found a positive association for detection of viruses in water sources with at least one latrine within a 50 m radius. They postulated that during the wet season viruses were transported in shallow groundwater, whereas in the dry season contamination was likely to be from surface water.

In an informal settlement in Zimbabwe, Zingoni et al. (2005) found detectable total and faecal coliforms in over two-thirds of domestic boreholes and wells. In the area, 75% of households used pit latrines and there were also informal trading areas. In Langas, Kenya, Kimani-Murage and Ngindu (2007) found that 50% of wells were within 30 m of a pit latrine and that all shallow wells were positive for total coliforms with 70% >1,100 mpn/100 ml; however, in Kisumu, Wright et al. 2013) failed to find a significant correlation between the levels of thermotolerant coliforms (TTC) in water sampled from shallow wells and the density of pit latrines.

#### Chemical contaminants

The chemical species of greatest concern from excreta disposed in on-site sanitation systems are regarded to be nitrate, phosphate and chloride. Pin-pointing specific sources is challenging as nitrate may be derived from numerous sources including plant debris, animal manure, solid waste and fertilisers. A common approach has been to compare areas that are similar but have different latrine densities. In an informal settlement in Zimbabwe, Zingoni et al. (2005) demonstrated that the highest nitrate concentrations were associated with the highest population and pit latrine density. A similar pattern was observed in Senegal and South Africa (Tandia et al. 1999; Vinger et al. 2012). Studies in the peri-urban areas of Kisumu, Kenya, have shown that the density of latrines within a 100 m radius of the sources was significantly correlated with nitrate levels (Wright et al. 2013). In eastern Botswana, the build-up of nitrogenous latrine effluent in soils and downwards leaching resulted in nitrate concentrations of above 500 mg/L (Lewis et al. 1980). The highest concentrations are found downstream of areas with high latrine use (Vinger et al. 2012). Similarly, Tijani and Onodera (2005) attributed high NO_3_ (>50–412 mg/L) in shallow groundwater system in Ibadan, Nigeria, to leachates inputs from in-house soak-aways and pit latrines. Direct measurements are sparse but Graham and Polizzotto (2013) estimate lateral travel distances of 1–25 m for pit-latrine derived nitrate. Sangodoyin (1993) found that nitrate concentrations were not related to distance from the source in Abeokuta, Nigeria.

Chloride is typically transported with minimal retention and frequently tracks nitrate (e.g. Lewis et al. 1980) unless subsurface conditions promote denitrification. Ammonium does not tend to accumulate in groundwater near latrines but can accumulate and persist in anaerobic conditions and when the water table intersects the base of the latrine pit (Ahmed et al. 2002; Dzwairo et al. 2006). Other contaminants include potassium, sulphate and dissolved organic carbon (DOC).

### Impacts from non-sanitary anthropogenic sources

Table 4 summarises studies focused on the impact of waste dumpsites, industrial activity and mining on groundwater quality. There are only a handful of case studies which have characterised the impacts of waste dumps and industry in urban areas and these are dominated by examples from Nigeria. Examples of contamination from historical mining activity come from Nigeria (Ezekwe et al. 2012) and Zambia (Nachiyunde et al. 2013a).

Oyeku and Eludoyin (2010) investigated contamination in wells and boreholes from industrial and waste dumps in Ojota, Nigeria. Sites within a 2 km radius were found to be affected with elevated concentrations of Fe, Cu, Pb and Zn as well as high SEC. In Akure, Nigiera, boreholes in the vicinity of a landfill were found to be contaminated, with high nitrate (36–61 mg/L), Pb and Zn (Akinbile and Yusoff 2011). In Lagos, Longe and Balogun (2009) found elevated concentrations of nitrate (mean 38.5 mg/L), phosphate (mean 10.7 mg/L) and ammonium (mean 0.2 mg/L) as well as high TDS (3–23 g/L) in wells between 10 and 400 m from a municipal landfill. Kassenga and Mbuligwe (2009) characterised seasonal and up/down gradient water quality in wells in the vicinity of a solid waste dump in Dar-es-Salam, Tanzania. Higher faecal coliforms (FC) counts were found in sites down gradient (3.4 × 10^4^–3.7 × 10^4^) compared to up gradient (1.5 × 10^4^–0.7 × 10^4^). Higher FC counts were also found during the wet season compared to the dry season down gradient of the dumpsite. Lower SEC as well as SO_4_ and Fe were found up gradient and in the wet season, implying dilution from rainfall and recharge and contamination from the dumpsite.

Elevated concentrations of Pb (0.24 mg/L), Cd (0.25 mg/L) and Zn (1.07 mg/L) were found in wells in the vicinity of mining activities in Nigeria (Ezekwe et al. 2012). A study by Nachiyunde et al. (2013a) included sites from the Copperbelt in Zambia and found overall low contamination levels in groundwaters with no trace elements above WHO drinking-water guideline values. von der Heyden and New (2004) also carried out a detailed study in the Zambian Copperbelt on the impact of mine tailings on groundwater quality. They found that there was only local effect from the tailings and that concentrations of Co, Ni and Zn were below WHO drinking-water-quality standards.

### Seasonal trends in groundwater quality and implications for climate change

There are relatively few studies that have undertaken regular water quality monitoring or have carried out seasonal comparisons. Studies by Howard et al. (2003) and Sorensen et al. (2015a, b) are two notable examples where detailed seasonal monitoring of microbiological indicators was carried out over a 12 month period to characterise the risk factors for spring contamination in Kampala, Uganda, and shallow wells and boreholes in Kabwe, Zambia. Significantly higher contamination has been observed after rainfall events and there was strong evidence that rapid recharge of the shallow groundwater causes a rapid response in spring and shallow well water quality (Barrett et al. 1998; Sorensen et al. 2015b).

Higher maximum faecal streptococcal counts were found in the wet season compared to the dry season for studies in Uganda (Kulabako et al. 2007) and Malawi (Palamuleni 2002). Changes in nitrate show a mixed picture with higher maximum concentrations in two studies from Uganda and DRC (Kulabako et al. 2007; Vala et al. 2011) during the wet season, while in the case study from Zimbabwe (Mangore and Taigbenu 2004) lower maximum values were found. Median values for nitrate were lower in the wet season for both the Uganda and Zimbabwe case studies (see Table S1 of the ESM), which may indicate a dilution effect, while the higher maximum concentrations may be explained as a result of a pulse of contaminants at the start of the rainy season and or evaporative effects during the dry season. Understanding seasonal trends in nitrate are complicated by the changes in redox conditions, particularly in low-lying areas which are prone to flooding in the wet season which are not uncommon in SSA, e.g. Lusaka, Zambia. These may shift from an oxidising regime during low water levels, which retains NO_3_, to a reducing regime where denitrification can take place during inundation (Sanchez-Perez and Tremolieres 2003; Spalding and Exner 1993).

Future climate scenarios for Africa are highly uncertain (Schmidhuber and Tubiello 2007; Conway et al. 2009) but some regions such as East Africa have been acknowledged as facing large risks from impacts of climate change (Hinkel et al. 2012). There are few studies that have been undertaken in Africa that provide direct evidence of the impact of climate change on deteriorating water quality and impacts on health; however, there is growing evidence globally of an association between water-borne diarrheal diseases and climatic factors that are likely to intensify under future climate change scenarios (Levy et al. 2016). Levy et al. (2016) suggest that these include risks associated with increased temperatures, more intense rainfall events and greater amounts of flooding. Several studies suggest there are greater risks of microbiological contamination due to either flooding conditions in African cities or due to the onset of the rainy season (Howard et al. 2003; de Magny et al. 2012; Sorensen et al. 2015a, b) and links between disease outbreaks and El Niño years have been proposed (de Magny et al. 2012; Olago et al. 2007). There is a clear need for climate appropriate sanitation and water provision in rapidly expanding urban centres (Howard et al. 2016). Any future increases in rainfall intensity may enhance groundwater recharge processes and may lead to more rapid degradation of water quality through enhanced transport of contaminants to the water table (Taylor et al. 2009). The impact of saline intrusion on fragile shallow coastal aquifers in Africa is already being observed (e.g. Comte et al. 2016) and will be compounded by any significant future rise in sea levels (Hinkel et al. 2012).

### Comparisons between different groundwater source types

Table 5 summarises and compares water quality data from studies where multiple sources have been sampled for comparison. Open and unlined wells are consistently of poorer quality compared to lined or ‘improved’ wells (e.g. Godfrey et al. 2006; Sorensen et al. 2015b). Boreholes generally have the best water quality, however in some studies springs have been found to be of better quality compared to boreholes (e.g. Takem et al. 2010) and in others cases the trend is reversed (e.g. Palamuleni 2002) or both sources were found to have comparable levels of contamination by FC (e.g. Abiye 2008). It is important to note that many of these studies contained very few observations for each source type and generalisations should be treated with caution; however, together they form a more compelling body of evidence.

**Table 5:** Comparison of microbiological water quality from multiple groundwater sources including boreholes, wells and springs (9/48)

Town/city/area	Country	Geology/sites	Water quality (cfu/100 ml)	Contamination	Reference
Oju area	Nigeria	Sedimentary *n* = 30	Borehole: FC BDL–500, typically <200. Improved well: FC 50–500, typically >200. Traditional well: FC > 500	Borehole < improved well < traditional well	Bonsor et al. (2014)
Yaounde	Cameroon	Basement *n* = 40	Spring: FC 2–72, FS 0. Well: FC 7–100, FS 0–100	Spring < well	Ewodo et al. (2009)
Kumasi	Ghana	Basement *n* = 9	Well: FC mean >30,000, EnC = 0–1,152. Borehole: FC mean > 20,000, EnC 0–36	Borehole < well	Obiri-Danso et al. (2009)
Blantyre	Malawi	Basement *n* = 9	Borehole: FC 0–30, FS 0. Spring: FC 530–9,500, FS 0–7,000. Wells: FC 3500–11,000, FS 250–2,650	Borehole < spring < well	Palamuleni (2002)
Njala	Sierra Leone	Basement *n* = 8	Spring: FC 50–30,000, FS 8–2500. Wells: FC 125–63,000, FS 5–2,500	Spring < well	Wright (1986)
Kampala	Uganda	Basement *n* = 16	Spring: FC 29–10,000, FS 6-8300. Wells: FC 0–26^6^, FS 0–26^8^	Spring < wells	Kulabako et al. (2007)
Harare	Zimbabwe	Basement *n* = 29	Borehole: FC 0–30,000. Well: FC 0–30,000	Borehole < well for FC	Zingoni et al. (2005)
Douala	Cameroon	Sedimentary *n* = 4	Spring: FC 1–950, FS 0–420. Borehole: FC 1–2,300, FS 0–1,400	Spring < borehole	Takem et al. (2010)
Kabwe	Zambia	Karstic *n* = 75	Borehole: FC<2–630. Well: FC <2–28,000	Borehole < well	Sorensen et al. (2015b)

*FC* faecal coliforms; *FS* faecal strep.; *EnC* enterococci, *TC* total coliforms, *BDL* below detection limit

There is some evidence that the water quality of wells may be affected by usage rates, i.e. with fewer groundwater sources being relied on towards the end of the dry season there is greater risk of contamination, e.g. from materials used for drawing water, especially for unimproved sources (Godfrey et al. 2006). For boreholes this contamination pathway is generally not a major risk factor and this supports the generally better quality found in these types of sources. The lower storage volume of shallow boreholes compared to wells may also be an important factor as this type of contamination can be more rapidly flushed out.

Improved wells do not generally exhibit the same level of gross contamination observed in traditional wells and springs; however, in the majority of studies, wells (both improved and unimproved) are found to have water with unacceptable levels of contamination with faecal coliforms by WHO standards (and typically > 100 cfu/100 ml) in at least some part of the year and often throughout the year. With perhaps the exception of highly karstic settings for microbiological and nitrate content, the following order of water source quality (best to worst) was found as follows: boreholes > improved wells = springs > traditional wells.

### Separation between sources of pollution and groundwater abstraction points

A number of studies carried out in Africa relevant to this topic are reviewed in the following section; most studies have focussed on minimum separations between pit latrines and wells. As part of their recent literature review, Graham and Polizzotto (2013) included an assessment of the minimum separation distances between pit latrines and groundwater receptors recommended by studies in a range of typical hydrogeological settings. Separation distances of 10–50 m were commonly recommended; however, there was no detailed consideration of higher-risk settings such as those posed by tropical soils, which cover a considerable part of Africa, or karstic settings which require considerably greater separation distances (e.g. Bonsor et al. 2014).

Microorganisms have been assumed not to survive for very long after excretion but recent studies with viruses suggest that water quality may be impaired for a considerable length of time. Using a mixture of routine culture methods and genetic detection methods, Charles et al. (2009) detected viruses over 300 days after they were introduced in simulated groundwater systems. Using similar survival time for viruses in groundwater systems in the Netherlands, Schijven et al. (2006) calculated that protection zones of between 1 and 2 years travel time would be required to ensure an infection risk of less than 1 in 10,000 per person per year. This is considerably longer than the 60-day travel time that is widely applied in Europe, or shorter in other parts of the world. Although a number of assumptions have been applied to the quantitative microbial risk assessments that were used to derive the travel time, it highlights the potential inadequacy of the current protection zones and points to the need for water treatment to ensure that it is safe to drink.

A number of approaches have been used to define the quantities and transport distances of latrine-derived microbial contaminants. The majority of these have been culture-based studies of faecal bacteria; but there has only been one study of viruses related to pit latrines (Verheyen et al. 2009).

Attenuation of microbes is likely to be dependent on the hydrological conditions both in terms of water levels and recharge rate and permeability of the aquifer, and is highly variable. Dzwairo et al. (2006) found faecal and total coliforms greatly reduced beyond 5 m from pit latrines in Zimbabwe, whereas Still and Nash (2002) found faecal coliforms to be attenuated to <10 cfu/100 ml after 1 m in Maputaland, KwaZulu-Natal. In Abeokuta, Nigeria, Sangodoyin (1993) found coliform attenuation to be correlated both with distance from the source and with the depth of the groundwater well. In Epworth, Zimbabwe, groundwater contamination was higher in the dry season rather than in the wet, with coliforms detected up to 20 m from the pit (Chidavaenzi et al. 1997). In Benin, Verheyen et al. (2009) found a positive association for detection of viruses in water sources with at least one latrine within a 50 m radius. They postulated that during the wet season viruses were transported in shallow groundwater.

While clearly less important from a health perspective compared to microbiological contamination, chemical contaminants also pose a threat to water quality, and they are very useful tracers of microbiological contaminants, which are inherently more transient in groundwater systems. Nutrients are also important in the fact that they are linked to the survival of pathogens in the environment.

Pin-pointing specific sources using nitrate is challenging as nitrate may be derived from numerous sources including plant debris, animal manure, solid waste and fertilisers. A common approach has been to compare areas that are similar but have different latrine densities. In an informal settlement in Zimbabwe, Zingoni et al. (2005) demonstrated that the highest nitrate concentrations were associated with the highest population and pit latrine density. Similar patterns have been observed in Senegal and South Africa (Tandia et al. 1999; Vinger et al. 2012). Studies in the peri-urban areas of Kisumu, Kenya, have shown that the density of latrines within a 100 m radius of the sources was significantly correlated with nitrate concentrations (Wright et al. 2013). In contrast, Sangodoyin (1993) found that nitrate concentrations were not related to distance from pit latrines in Abeokuta, Nigeria. In eastern Botswana the build-up of nitrogenous latrine effluent in soils and vertical leaching resulted in nitrate concentrations of above 500 mg/L (Lewis et al. 1980).

Direct measurements and well-designed studies are sparse and rarely consider rapid flowpaths or multi-point sources of contamination. Graham and Polizzotto (2013) estimate lateral travel distances of 1–25 m for pit-latrine derived nitrate. Chloride is typically transported with minimal retention and frequently tracks nitrate (e.g. Lewis et al. 1980) unless subsurface conditions promote denitrification. Ammonium does not often accumulate in groundwater near latrines but can accumulate and persist in anaerobic conditions and when the water table intersects the base of the latrine pit (Dzwairo et al. 2006; Nyenje et al. 2013). Other contaminant tracers of wastewater or faecal sources include potassium, sulphate and DOC and emerging organic contaminants (Sorensen et al. 2015c).

Proposing a single separation value is not realistic given the complexity of recharge and lateral/vertical pathways in different hydrogeological settings in SSA. At the very least, a consideration of soil permeability characteristics and the presence of laterites, as well as other macro-pore flow paths, need to be included as part of an assessment of safe separation distances. The separation distance approach has an inbuilt assumption about point sources such as pit latrines, as the dominant sources of contamination, and while this may be the case for many settings, more diffuse sources such as open defecation and animal waste need to be considered (Lapworth et al. 2015). Woodhouse et al. (1996) investigating safe water environments in Eldoret, Kenya, illustrate the prospects and problems of a holistic research method and take due cognizance of social dimensions, behaviour patterns and attitudes in the problem of groundwater pollution. The findings emphasized the importance of the multidimensional nature of safe water and other health issues in peri-urban areas as well as collaboration between anthropologists, medical practitioners, microbiologists, policy-makers and implementers such as local authorities and residents in addressing the issues.

### Assessing urban groundwater pollution risk and nitrate concentrations in SSA

**Fig. 3:**
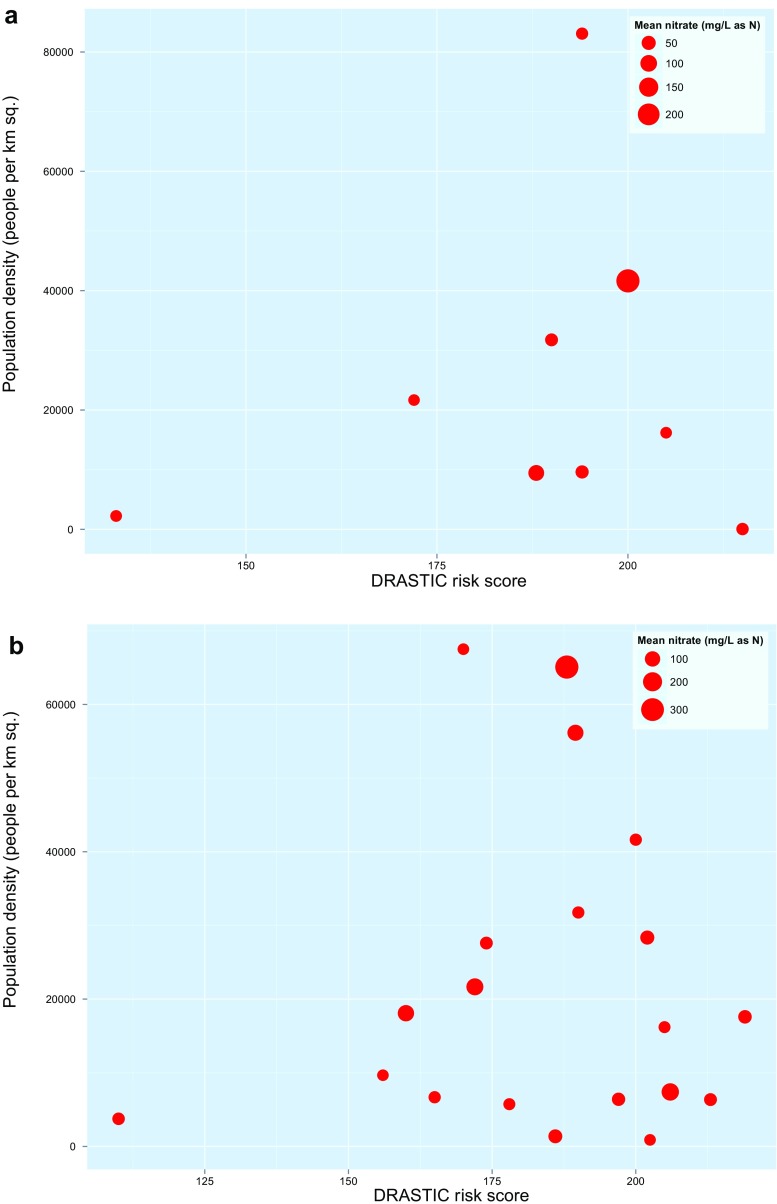
Relationship between mean nitrate levels, population density and DRASTIC aquifer vulnerability risk score among 31 studies that** a** predominantly sampled boreholes and** b** predominantly sampled wells and springs

Figure 1 shows the spatial distribution of the 31 studies included in the analysis of nitrate in urban groundwaters. Nigeria was a particular focus for studies of urban groundwater contamination with nitrate. Figure 3 shows mean nitrate recorded per urban groundwater study, compared with the DRASTIC risk score for underlying aquifer vulnerability (from Ouedraogo et al. 2016) and maximum population density in the settlement studied. Although the greatest nitrate contamination was in groundwater sources in settlements with extremely high population densities (over 40,000 people per km^2^), there was wide variation in study findings from aquifers with similar risk levels. Table 6 shows the results of the meta-regression of these risk factors for nitrate contamination.

**Table 6:** Results of a random effects meta-regression of risk factors for mean nitrate contamination in 31 studies of urban groundwater sources

Variable	Coefficient (95% confidence intervals)	*P* value
Aquifer vulnerability (DRASTIC) risk score	0.085 (0.077–0.093)	<0.001
Population density per ha	−0.00018 (−0.0015–0.0012)	0.79
Proportion of boreholes sampled	−0.105 (−0.346–0.136)	0.393
Constant	−13.374 (−14.835 to −11.913)	<0.001

This analysis extends a recent evaluation of nitrate contamination in relation to a continental-scale aquifer vulnerability index (Ouedraogo et al. 2016). Unlike that recent study, the analysis described in this paper differentiates boreholes from shallower groundwater sources, incorporates one measure of nitrate load into the aquifer (population density), and explicitly accounts for sample size variation between studies via random effects meta-regression. The findings suggest that mean nitrate contamination in included studies is highly variable and difficult to predict. This is likely to be a result both of variation in sampling protocols between studies and the coarse spatial resolution of the aquifer vulnerability and population density data used; however, meta-regression (Table 6) identified a significant relationship between the aquifer vulnerability index and mean observed nitrate, supporting the findings of Ouedraogo et al. (2016).

## Conclusions and research priorities

Over the last three decades there has been a concerted effort to develop improved groundwater supply and sanitation across Africa (Bartram and Cairncross 2010); however, the combination of limited waste management and high intrinsic vulnerability in many regions leave urban aquifers highly impacted by faecal and chemical loading, posing a clear health risk to users. Shallow groundwater sources, used widely for a range of domestic activities including drinking and cooking, are particularly at risk and are often the go-to water source in low-income areas in SSA.

While shallow urban supplies have high health risks, they remain an important source of water for millions of low-income urban communities across Africa, and due to cost, poor reliability of piped sources and in some cases due to taste, are often preferred to treated piped supplies. Despite a growing dependence on urban groundwater resources, this paper shows that compared to other regions globally there have been relatively few systematic urban groundwater quality studies carried out in SSA. This is due to a combination of factors including the costs associated with water quality monitoring, the relatively low levels of funding for research in SSA over the past decade, limited national regulation of groundwater in many cases and the relatively small number of groundwater professionals in this region. Key conclusions from this paper are:

- There is a paucity of high-quality studies and limited systematic monitoring data for groundwater quality in SSA. More systematic studies, including those with larger populations and randomized designs and therefore more generalizable, as well as focussed high-frequency temporal studies, are needed to provide a better evidence base from which to make recommendations for groundwater protection and improved health outcomes in SSA.
- Sources of faecal contamination, many of which can by-pass natural attenuation mechanisms, are widespread in most urban centres in SSA, posing a continued threat to shallow groundwater supplies and users.
- High-intensity rainfall events pose a risk to shallow and poorly protected groundwater sources, this is particularly an issue for unimproved dug wells and springs. Shallow groundwater levels also pose a significant risk to shallow water sources in urban settings due to reduced attenuation in the unsaturated zone when groundwater intersects with the base of pit latrines and sewer networks.
- Borehole sources, and the deeper (e.g. > 40 mbgl) groundwater resources they often access, are of generally much better chemical and microbiological status compared to shallow groundwater sources, and as such these sources need to be protected and managed. However, contamination may still be a major issue for boreholes in karstic or fractured basement settings or where there has been long-term contaminant loading to the subsurface and/or high rates of abstraction.
- Results from the literature suggest that using fixed separation distances between sources and receptors cannot be applied without considering the detailed hydrogeological setting, including an understanding of rapid lateral and vertical pathways that may be present, and assessing risks from diffuse sources of contamination.
- There is some evidence (Table 6) to support a link between DRASTIC scores and mean nitrate levels in urban areas reported in literature. However, this relationship is complex due to a range of factors including the variety of urban and non-urban nitrate sources of pollution as well as hydrogeological N processes that may remove nitrate, as a result there are limited benefits from using this approach to understand urban nitrate pollution in groundwater.

Important knowledge gaps remain for understanding of rapid pathways for aquifer contamination, which are important for assessing microbiological contamination, and in some cases can even impact deeper groundwater sources. For the majority of urban areas in SSA there is a paucity of information on the quality and quantity of the groundwater resource, particularly in terms of vertical permeability characteristics and local flow patterns and the response to intense rainfall events. So far, a relatively limited range of groundwater contaminants has been reported in the literature for SSA and there is little or no systematic monitoring of key microbiological and chemical drinking-water-quality parameters. Given the potentially important role rapid horizontal (and vertical) pathways play in tropical soils overlying basement aquifers (e.g. Bonsor et al. 2014) on the migration of contaminants in the subsurface, and the widespread occurrence of this aquifer type across SSA, this is a key topic that warrants further investigation. Although not the focus of this paper, it is clear that socio-economic aspects need to be better integrated with physical hydrogeology if urban groundwater security research is to be effective and relevant.

Tracing and quantifying pathogen residence times/survival, sources and speciation in the subsurface is a clear research priority. Assessments in shallow groundwater systems as well as deeper systems are required to make a robust assessment of the vertical and lateral separation required between sources of pollution and strategic groundwater resources. New techniques such as molecular marker methods and qPCR techniques (e.g. Mattioli et al. 2012; Sorensen et al. 2015a) for fingerprinting pathogens as well as new fluorescence sensor technology for rapidly mapping microbiological contamination of water sources (e.g. Sorensen et al. 2015b) and other field tests may offer new ways to discriminate between different pathogen sources and pathways for contamination and rapidly assess potential health risks associated with vulnerable groundwater sources.

Given the high natural variability in rainfall patterns and potential intensification of future climate patterns across some parts of Africa, there are a number of climate-related groundwater-quality research priorities, which include: the link between rainfall intensity and enhanced groundwater recharge and rapid water-quality deterioration within vulnerable shallow urban aquifer systems; the impact of increasing temperature on pathogen occurrence and survival in the urban environment, particularly during urban flooding; enhanced risks of pit latrine flooding and groundwater contamination during high intensity rainfall events in low-income areas; the impact of rising sea levels and saline intrusion on fragile shallow coastal aquifers given the large number of growing coastal urban centres across Africa. Priority regions for research are areas which are experiencing the fastest urban population growth and expansion, and include regions such as the Lake Victoria Basin and parts of West Africa (UN 2015; UNEP 2008).

Targeted investments in priority research areas, including those highlighted in the preceding, are needed in SSA to provide evidence to inform the provision of climate ready urban water and sanitation infrastructure. This is required in order to enhance safe drinking water and sanitation delivery to the rapidly expanding urban and peri-urban populace and work towards the sustainable development goals in SSA. Moreover, with ongoing health epidemics in urban areas, including viral/bacterial diseases, many of which are from faecal sources, both the national governments and international community should consider investments in improved waste management and clean and safe water supplies as a matter of priority.

## Electronic supplementary material

Below is the link to the electronic supplementary material.

ESM 1 (PDF 926 kb)
