# Supplementary material for: Urban groundwater quality in sub-Saharan Africa: current status and implications for water security and public health
Source: Hydrogeol J. 2017 Jan 18;25(4):1093–116. doi: 10.1007/s10040-016-1516-6 (PMC6991975; doi:10.1007/s10040-016-1516-6)
Supplement: Supplementary file 1 — (PDF 926 kb) [file 10040_2016_1516_MOESM1_ESM.pdf]

Urban groundwater quality in sub-Saharan Africa: current status and implications for water security and public health

Hydrogeology Journal – Electronic Supplementary Material

Lapworth D.J[1]*, Nkhuwa D.C.W[2], Okotto-Okotto J[3], Pedley S[4], Stuart M.E[1], Tijani M.N[5], Wright J[6]

[1] British Geological Survey, Maclean Building, Wallingford, OX10 8BB, UK

[2] University of Zambia, Great East Road Campus, P.O. Box 32379, Lusaka, Zambia

[3] Victoria Institute for Research on Environment and Development (VIRED) International, Rabuour Environment and Development Centre, Kisumu-Nairobi Road, P.O. Box 6423-40103, Kisumu, Kenya

[4] Robens Centre for Public and Environmental Health, University of Surrey, Guildford, GU2 7XH, UK

[5] Department of Geology, University of Ibadan, Ibadan, Oyo State, Nigeria

[6] Geography and Environment, University of Southampton, Highfield, Southampton SO17 1BJ, UK

*Corresponding author, email: djla@bgs.ac.uk

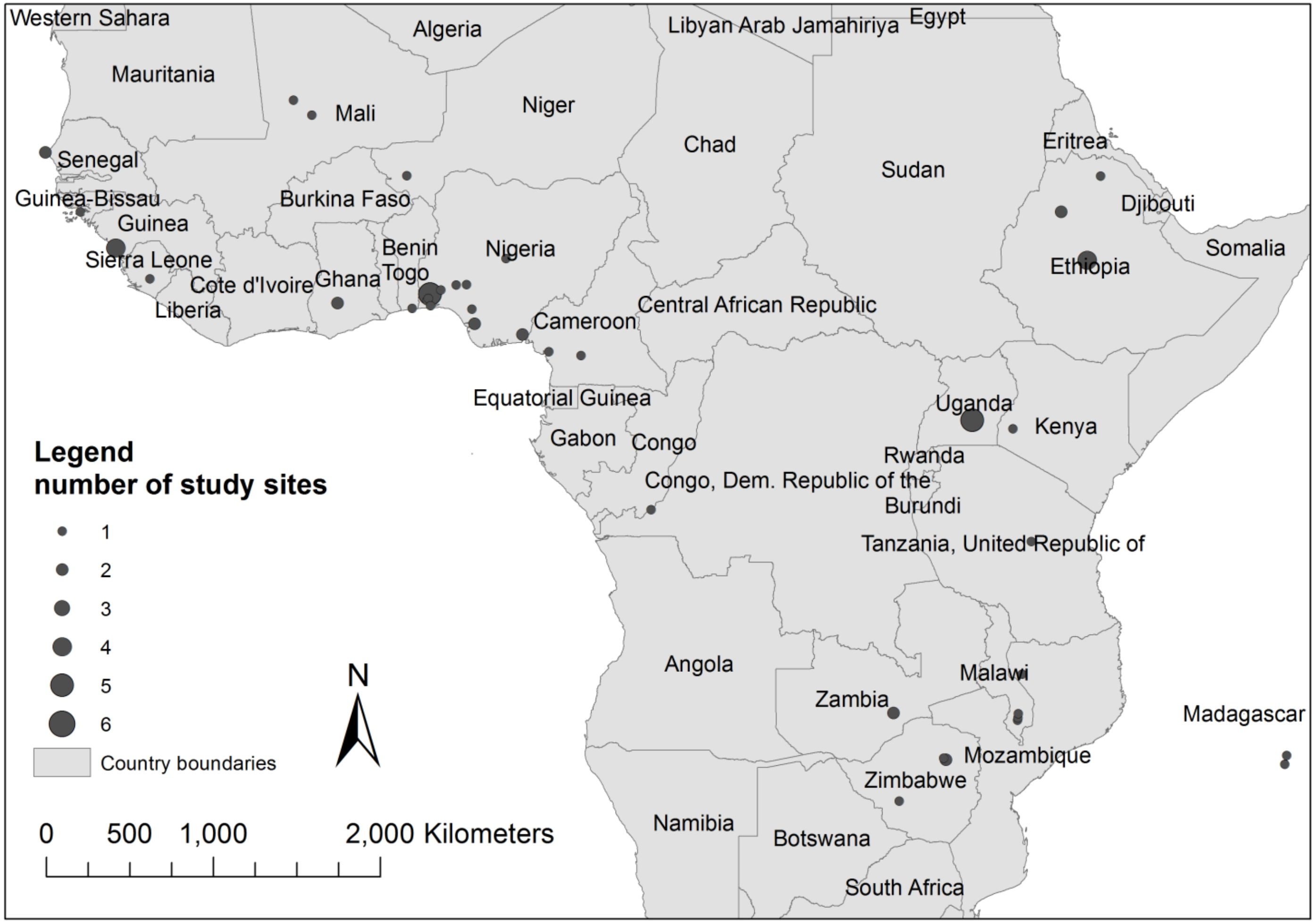

Figure S1 **Locations of the 31 studies included in the meta-analysis of nitrate concentrations of urban groundwater in sub-Saharan Africa (31 of the 48 of studies listed in Table S1)**

Table S1 Results from the literature review of urban groundwater quality studies across sub-Saharan Africa (*n*=48)

Area	Geology	Sample sites (*n*)	Results from selected water quality parameters		Sampling time-frame	Conclusion and sources of contamination	Reference
[2]Dakar, Senegal	Quaternary sediments	Wells (56)	NO_3 0-122		July-October 1997	Nitrate contamination from point-source seepage in urban areas	Cissé Faye et al. (2004)
[2]Bolama City, Guinea Bissau	Sandy soils and Cenozoic –Modern sediments	Wells (28)	SEC 27-326, mean 136<br>Turb. 1-26, mean 6.5<br>TC 0-23000, mean 2306<br>FC 0-5000, mean 410<br>Fecal Enterococci 0-850, mean 74<br>NO_3 0.9-55.3, mean 16.6<br>NH_4 0.01-1.37, mean 0.11<br>NO_2 0.03-0.13, mean 0.04<br>Cu, Fe, Cr, As,		July 2006	80% of wells contaminated with FC linked to widespread use of PL	Bordalo and Savva-Bordalo (2007)
[3]Conakry, Guinea	Volcanic rocks, fissured	Wells (69)	**Mod. wells**<br>FC 370-1x10^5<br>FS 90-9k<br>NO_3 2-46<br>NH_4 0.06-7<br>Cl 17-130<br>F 0-0.16<br>Turb. 1-70	**Trad. wells**<br>FC 50-2 x10^5<br>FS 150-2 x10^4<br>$NO_3$7-51<br>NH_4 0.01-8<br>Cl 8-284<br>F 0.0.38<br>Turb. 1-63	Dry season April-May 1994	Widespread contamination by nitrate and FC linked to poor sanitation and well construction	Gélinas et al. (1996)

Area	Geology	Sample sites (*n*)	Results from selected water quality parameters	Sampling time-frame	Conclusion and sources of contamination	Reference
[3]Bo, Sierra Leone	Granitic Basement	Wells (33) lined and unlined	FC 0-75, mean 19.6<br>NO_3 0.5-28, mean 7.7<br>PO_4 0.01-11.5, mean 1.7<br>SEC 39-1281, mean 362	Wet season	Distance from field significant predictor of FC, not distance from toilet/PL	Jimmy et al. (2013)
[2]Various. Ivory coast	Basement	Boreholes (230)	NO_3 mean 69	1981 and 1982	High nitrate (up to 200 mg/L) linked to domestic pollution and deforestation	Faillat (1990)
[1]Kumasi, Ghana	Precambrian Basement	Hand-dug wells (10)	TDS 6-230, mean 113<br>NO_3 0-0.968, mean 0.16<br>PO_4 0.67-15, mean 7.8<br>TH 8-103, mean 54<br>TC and E. coli <20	N/A	Water quality survey showed that water quality parameters were within WHO drinking water guideline values	Nkansah et al. (2010)
[3]Kumasi, Ghana	Precambrian Basement	Borehole and wells in peri-urban communities (9)	Fe 0.001-0.955<br>Mn 0.018-0.238<br>Pb 0.005-0.074<br>TC 3-16.8×10^6<br>FC 1.5-4.37×10^4<br>Enterococci 1.3-53.5	Monthly between Dec 2000 and Jan 2001	Poor quality overall, contamination linked to proximity to PL and refuse tips as well as livestock	Obiri-Danso et al. (2009)
[2]Cotonou, Benin	Quaternary to mid Pleistocene sandstone	Dug wells in upper aquifer in densely populated area (379)	SEC 320-1045<br>Mn 0.06-0.19<br>NO_3 10.4-118<br>PO_4 <0.05-21.6<br>SO_4 3.14-86.3	May 1991, August 1991 and April 1992	High P and K concentrations in upper aquifers linked to anthropogenic pollution	Boukari et al. (1996)
[1] Ougadougou, Burkina Faso	Basement	>1000 sites, Time series for nitrate and SEC in groundwater	pH, SEC and NO_3<br>Nitrate:<br>**Wells** <50-250<br>**Boreholes** 0-150	2001-2004	Nitrate concentrations higher during high rainfall conditions. Shallow wells have higher NO_3 concentrations compared to boreholes	Ouandaogo-Yameogo et al. (2013)

Area	Geology	Sample sites (*n*)	Results from selected water quality parameters	Sampling time-frame	Conclusion and sources of contamination	Reference
[3]Ilesha, Nigeria	Basement	Wells (86)	Mean results:<br>NO_3 35<br>Cl 34<br>SO_4 2.8	Single survey	Evidence of anthropogenic impact on water quality degradation using PCA	Malomo et al. (1990)
[1]Benin City, Nigeria	Quaternary to mid Pleistocene sandstone	Boreholes and open wells (6)	Pb 0.03-0.25<br>Zn 0.98-7.19<br>Cr 0.02-1.1<br>Cd Nd-0.23<br>FC 4600-240000<br>FS 600-35000	Single survey	Elevated Pb, Cr, Cd and Zn attributed to indiscriminate waste disposal and FC occurrence linked to PL, soak-always and septic tanks	Erah and Akujieze (2002)
[2]Calabar, Nigeria	Tertiary to recent sands and gravels	Existing wells (20)	BOD 0.06-4.09, mean 1.72<br>N 0.09-3.5, mean 2.15<br>Cl 0.1-1, mean 0.45<br>FC 0.75-4.32, mean 1.86	N/A	FC, nitrate and Cl had a positive correlation with urbanisation	Eni et al. (2011)
[1]Ibadan, Nigeria	Basement, banded gneiss and schist	Existing wells (N/A)	TSS 159-186.6, mean 174<br>Cl 1.1-10, mean 5<br>TC 2300-9200, mean 5120	Dry season	Gross pollution of groundwater attributed to poor well construction, PL and waste management	Ochieng et al. (2011)
[2]Ibogun, Pakoto, Ifo, Ogun State, Nigeria	Cambrian basement geology and weathered regolith	Dug wells, communities of 5000-20,000 people (20)	TDS 100-2200<br>TH 6-246<br>NO_3 0.8-88<br>TC 0-0.6 (cfu x10^5)<br>FC 0-0.2 (cfu x10^5)<br>FS 0-0.7 (cfu x10^5)	July-August 2009	Water quality standards for nitrate, FC, FS not met for significant proportion of wells	Adelekan (2010)
[1]Lagos, Nigeria	Alluvium over sedimentary	Urban wells (18)	TDS 79-1343, mean 514<br>TH 24-289, mean 110<br>Na 8-274, mean 79	Survey August to October 2004	Sources of contamination included sanitation, textiles,	Yusuf (2007)

Area	Geology	Sample sites (*n*)	Results from selected water quality parameters	Sampling time-frame	Conclusion and sources of contamination	Reference
			NO_3 0.05-1.51, mean 0.4<br>Pb 0-1.9, mean 1.6<br>Zn 0-4.2 mean 0.3		pharmaceuticals, food, tanneries, motor industry	
[1]Surulere, Lagos, Nigeria	Alluvium over sedimentary	Wells and boreholes in a middle class area (49)	Al 1-99 µg/L<br>Cd 1-98 µg/L<br>Pb 1-24 µg/L	July 2009	Pb and Cd above WHO drinking water standards in >30% of sites	Momodu and Anyakora (2010)
[1]Abeokuta, Nigeria	Basement igneous and metamorphic	Shallow wells including sanitary survey (40)	All bacterial count>20<br>Maximum 800 E. coli +PA+SAL	December 2005	Shallow groundwater is highly contaminated with bacteria. Sources include pit latrines, livestock and solid waste	Olabisi et al. (2008)
[2]Abeokuta, Nigeria, urban & peri-urban	Basement igneous and metamorphic	Shallow wells (76)	**Urban** (mean)<br>TDS 402<br>TH 30.3<br>NO_3 12.02<br>PO_4 0.21<br>Pb 0.25<br>Zn 0.12<br>TC 10500<br><br>**Peri-urban** (mean)<br>TDS 263<br>TH 31.7<br>NO_3 10.7<br>PO_4 0.03<br>Pb 0.19<br>Zn 0.09<br>TC 10000	Dry season	Mean values for Pb, nitrate E. coli and TC > WHO standards. Trading, textiles, transport, cottage industries, pit latrines<br>Generally higher in dry season	Orebiyi et al. (2010)
[1]Peri-urban area, Abeokuta, Nigeria	Basement igneous and metamorphic	Hand-dug wells (25)	TDS 50-270, mean 163<br>NO_3 2.97-40.7, mean 17.6<br>NH_4 0-0.59, mean 0.11<br>PO_4 12-86 µg/L , mean 46<br>TH 12-210 , mean 106	Rainy season 2008	Direct surface run off into wells is suggested as possible contamination source	Taiwo et al. (2011)

Area	Geology	Sample sites (*n*)	Results from selected water quality parameters	Sampling time-frame	Conclusion and sources of contamination	Reference
[1]Warri River plain, Delta, Nigeria	Alluvial Benin formation	Boreholes near WW treatment plant	TDS 16-81<br>COD 0.4-44.4<br>NO_3 0.3-1.2<br>Fe 0.05-0.15	2 year sampling campaign	River infiltration, municipal wastewater, agriculture, oil industry	Ibe and Agbamu (1999)
[1]Warri River plain, Delta, Nigeria	Quaternary and older sedimentary sequences	Dug wells	Fe 0.32-2.75<br>Pb 0.058-0.443<br>Ni 0.008-0.188<br>V 0-4<br>Cr 0-9<br>Cd 0.75-8.5<br>Zn 0-1.8	N/A	Sources include Warri River, settlement, refinery. Highest values in village 3 km from refinery	Aremu et al. (2002)
[1]Masaka, Nigeria	Cretaceous sandstone and clay	Dug wells, high density (12)	TDS 528-935<br>NO_3 44.5-92.5<br>Alk 67-179<br>Cl 41-118<br>Fe 0.085-0.199<br>Cr 0.005-0.0126<br>TC 25900-78400	Samples taken in wet season	WHO standards exceeded for a range of contaminants including nitrate, TDS, Cr, Cd and TC. High density settlement with shallow water table	Alhassan and Ujoh (2011)
[2]Yaounde, Cameroon	Basement	Springs and wells in high density area (> 40)	SEC 18.2-430, mean 87<br>FC 60% >100<br>FS 5%>100	One-off survey	Groundwater's in high density zones show significant degradation (chemical and microbiological), linked to PL	Ewodo et al. (2009)
[2]Douala, Cameroon	Alluvium over Pliocene sand and gravel	Springs , wells and boreholes (72)	SEC 25-362<br>NO_3 0.21-94.3<br>FC 0-2311	One-off survey	High levels of FS indicative of contamination from PL, related to age and density of settlement	Takem et al. (2010)
[2]Brazaville, DR Congo	Alluvial and sedimentary sequences	Wells	TDS 110 +/- 52.1<br>NO_3 1.32 +/- 0.64	Seasonal sampling	Low nitrate concentrations and low TDS levels	Matini et al. (2012)

Area	Geology	Sample sites (*n*)	Results from selected water quality parameters		Sampling time-frame	Conclusion and sources of contamination	Reference
[2]Kinshasa, DR Congo	Alluvial and sedimentary sequences	Wells including sanitary survey	**Dry season**<br>TDS 180-450<br>NO_3 76-118<br>PO_4 0.53-4.6<br>TH 110-149<br>Pb 0.04-0.09<br>Cd 0.13-0.20	**Wet season**<br>TDS 200-710<br>NO_3 97-198<br>PO_4 3.6-14.6<br>TH 17-52.5	One-off survey	Latrines, metal works, solid waste dumps are main sources of contamination	Vala et al. (2011)
[2]Mekelle, Ethiopia	Mesozoic sediments	Wells, springs and boreholes (100)	SEC 542-5300<br>TDS 330-3454<br>NH_4 0.01-2.38<br>NO_3 0.21-336<br>Cl 5.76-298<br>F 0-1.27, PO_4 0.001-0.58		N/A	Highly variable water quality indicative of a range of redox zones and sources of contamination	Berhane and Walraevens (2013)
[2]Bahir Dar, Ethiopia	Weathered and fractured Alkaline Basalt	Dug wells and protected pumps in inner, middle and outer zones (8)	**Middle and inner city**<br>TDS 20-600<br>NO_3 0.18-57.2<br>NH_4 0-12<br>Cl 46-270<br>FC 93% of sites<br>Mean 1.5 log cfu<br>E. coli 80% sites<br>mean 1.4 log cfu	**Outer city**<br>TDS 20-70<br>NO_3 0.08-8.8<br>NH_4 0-12<br>Cl 0-40	Sampling over a 5 month period 2006/2007	Groundwater contamination linked to population density and urbanisation. All dug wells and boreholes had microbiological contamination in excess of WHO/EU standards. Dug wells had significantly higher FC.	Tabor et al. (2011)
[1]Addis Ababa, Ethiopia	Volcanics	Boreholes and springs (9)	Alk 8-41<br>NO_3 0.72-35<br>NO_2 <0.01<br>COD 6.8-41<br>Cl 6.8-28		Various	The authors made a link between the surface water quality and groundwater quality. Major sources of contamination inferred were domestic waste, and industrial pollution from	Abiye (2008)

Area	Geology	Sample sites (*n*)	Results from selected water quality parameters	Sampling time-frame	Conclusion and sources of contamination	Reference
			PO_4 <0.03-0.1<br>Pb 4.6-25 μg/L<br>SEC 300-1200<br>TC 0-34000		textile industry and petrol stations	
[1]Addis Ababa, Ethiopia	Volcanics	Springs and boreholes (10)	Zn 0.87-146 μg/L<br><br>Ni 0.31-0.98 μg/L<br>Cu 0.44-1.82 μg/L<br>Pb 4.3-56.2 μg/L<br>Cd <0.1-0.2 μg/L<br>Co <0.1-0.12 μg/L	2002	Geogenic sources of heavy metals is the likely sources of groundwater contamination in this setting due to high heavy metal concentrations in soils and rocks	Alemayehu (2006)<br>Goshu and Akoma (2011)<br>Goshu et al. (2010)
[1]Addis Ababa, Ethiopia	Volcanics	Springs and wells (63)	Ni 2-152 μg/L<br>Pb <1 μg/L<br>Co 0.5-165 μg/L<br>As <3 μg/L<br>Zn <20-2100 μg/L<br>Cu 1.5-164 μg/L<br>Cd 0.3-12.3 μg/L<br>Cr 18.2-214 μg/L	Februrary-March 2004, July to September 2005	Urban area, leaching from polluted soils.	Demlie and Wohnlich (2006)
[1]Wajir, Kenya	Limestone	Wells (30)	NO_3 >45 in 50% sites<br>E. coli >50 for 50% sites, >100 for 35% of sites	Single campaign	Widespread pollution of shallow wells with nitrate and EC. Deeper aquifers are not suitable due to high salinity and low yields.	Mailu (1997)
[2]Eldoret, Kenya	Tertiary Volcanics	Shallow wells (31)<br>Boreholes (4)<br>Tap water (5)	TTC<br>Wells: all but one site had >1100<br>Boreholes: <DL-23<br>Tap: <DL	Single campaign	40% of shallow wells wer <15 from a PL, 54% were 15-30 from a PL	Kimani-Murage and Ngindu (2007)

Area	Geology	Sample sites (*n*)	Results from selected water quality parameters		Sampling time-frame	Conclusion and sources of contamination	Reference
[3]Kisumu, Kenya	Sedimentary/ Volcanics	Existing wells (191)	TTC 0->100k mean 894<br>NO_3 0.06-45 mean 15<br>Cl 0-225 mean 796<br>F 3-29.6 mean 6.2		1998 and 2004	Density of PL within a 100 m radius was significantly correlated with nitrate and Cl but not FC (*PC*)	Wright et al. (2013)
[2]Lichinga, Mozambique and Timbuktu, Mali	Quaternary/ Basement gneiss-granite complex	Hand dug wells: Timbuktu(31), Lichinga (159)	**Timbuktu**<br>SEC 221-2010<br>NO_3-N 35 med<br>Cl 500	**Lichinga**<br>SEC 220 med<br>NO_3 5.6 med<br>Cl 13.5	Timbuktu September 2002 to May 2003 Lichinga, April 2002-August 2004	Contamination of groundwater sources from on site sanitation traced using N:Cl	Cronin et al. (2007)
[3]Lichinga, Mozambique	Mudstone	Lichinga (25)	TTC, and Enterococi		Monthly for 1 year	Higher risk at onset of the wet season and end of the dry season. Predominant source was from animal faeces rather than PL or septic tanks. (LR)	Godfrey et al. (2006)
[2]Kampala, Uganda	Weathered Basement	Wells and springs	**High density**<br>NO_3 mean 67<br>Cl mean 59<br>TC mean 14	**Low density**<br>NO_3 mean 22<br>Cl mean 21<br>TC mean 544	Contrasting hydrological conditions	Significantly higher contamination in high density regions compared to low density	Barrett et al. (1998)
[3]Kampala, Uganda	Weathered Basement	Springs (25)	TTC (FC)<br>FS BLD-23000		Monthly between September 1998-March 1999	Evidence of rapid recharge to springs following rainfall. Local environment hygiene and improved sanitary completion shown to be more important than on-site sanitation for spring protection (LR)	Howard et al. (2003)

Area	Geology	Sample sites (*n*)	Results from selected water quality parameters	Sampling time-frame	Conclusion and sources of contamination	Reference
[1]Kampala, Uganda	Weathered Basement	Boreholes and wells (28)	Limited inorganic and organic suit, no microbiology	September and October 2011	Nitrate concentrations suggest poor sanitation and diffuse contamination.	Nachiyunde et al. (2013)
[3]Uganda, Kampala (urban)	Weathered basement	Piezometers (10)	1.5 m down gradient of pit latrines<br>NO_3 5-90<br>Cl 50-1100<br>$PO_4$0.1-2<br>NH_4 5-40	March-August 2010 biweekly sampling	PL found to be a significant source of nutrients (N) compared to waste dump. NH_4 removal by nitrification	Nyenje et al. (2013)
[1]Lusaka, Zambia	Dolomite	Wells and streams in intensely urbanised area (9)	SEC 200-710<br>NO_3 <0.1-43<br>NH_4 <0.25-3.5, Cl 4.6-36<br>PO_4 <0.1-4, B <1-10, As <0.2-0.49<br>Pb 0.14-0.67, Hg <0.4-13	July 2001	Values for nitrate and Hg were in excess of WHO standards on some occasions. Poor sanitation and solid waste disposal implicated.	Cidu et al. (2003)
[2]Lusaka, Zambia	Dolomite	Boreholes (7)	FC 0-45<br>TC 0-58<br>SEC 401-1060	Single survey	Evidence for contamination in health centre boreholes by FC, poor waste management implicated	Nkhuwa (2008)

**Area**	**Geology**	**Sample sites (*n*)**	**Results from selected water quality parameters**		**Sampling time-frame**	**Conclusion and sources of contamination**	**Reference**
[3]Lusaka, Zambia	Dolomite	Private and public boreholes (N/A)	Alk 124-564, NO$_3$ 0.03-39, NO$_2$ 0.002-42, NH$_4$ 0.08-60<br>Cl 42-102, TC 1-TNTC<br>FC 21-TNTC, BOD 2-69<br>COD 9-320		Various: 1995-2000	Hydrochem, microbiology and incidence of cholera outbreaks compiled to show the rapid deterioration of GW sources associated with poor sanitation	Nkhuwa (2003)
[2]Ndola, Zambia	Dolomite and basement lithologies	Wells (123) and boreholes (60) surface waters (41)	**Wells (median)**<br>TC 7<br>Zn 11.4 μg/L	**Boreholes (med)**<br>TC 0<br>Zn 139 μg/L	April-June 2013	Geological control on trace metal contamination. TC for wells>boreholes but no FC data collected.	Liddle et al (2015)
[3]Kabwe, Zambia	Dolomite and basement	Private (13) and public (12) boreholes, private wells (57)	**Dry season***Wells*<br><br>TTC 10-6800 (180)<br>Boreholes<br>TTC <2-28 (<2)	**Wet season**<br>*Wells*<br><br>TTC 2-27600 (570)<br>Boreholes<br>TTC <2-760 (<2)	Dry and wet season 2013-2014	Widespread FC contamination in shallow wells in both wet and dry seasons, wet>>dry. Generally good quality in peri-urban boreholes but evidence of contamination in some urban boreholes	Sorensen et al., (2015b)
[3]South Lunzu, Blantyre, Malawi	Weathered basement	Borehole, springs and dug well (9)	**Dry season**<br>SEC 210-330<br>Cl 21-35<br>Fe 0.1-0.8<br>FC 0-5200<br>FS 0-640	**Wet season**<br>SEC 306-383<br>Cl 14-29<br>Fe 0.4-0.7<br>FC 0-11,000<br>FS 0-7000	Wet and dry season on two occasions	Groundwaters highly contaminated due to poor sanitation and domestic waste disposal. 58% of residence use traditional PL	Palamuleni (2002)

Area	Geology	Sample sites (*n*)	Results from selected water quality parameters	Sampling time-frame	Conclusion and sources of contamination	Reference
[2]Tamatave and Foulpointe, Madagascar	Weathered basement and unconsolidated sediments	Boreholes (53)	FC 73%>0, 55% 0-10, 54%>10<br>NO_3 4.4-35, mean 23<br>Pb 1-215 μg/L, mean ca. 5 μg/L	One-off survey	Widespread drinking water contaminated with FC and concerns over Pb from pump materials	MacCarthy et al. (2013)
[3]Epworth and Harare, Zimbabwe	Granite	Wells and boreholes, transect of formal and informal zones (18)	NO_3 0-30, mean 11<br>PO_4 0-27.2, mean 3.03<br>FC 0-2, mean 0.75 (cfu x10^4)	Survey carried out with duplicate sampling	Pit latrines, faecal coliforms in older and informal trading areas, urban agriculture, home industries and commercial areas	Zingoni et al. (2005)

All concentrations are in mg/L unless otherwise stated, nitrate reported as N. SEC=specific electrical conductivity, PCA=Principal component analysis, LR= logistic regression, Alk=Alkalinity, TDS= total dissolved solids, TH=total hardness, BOD=biochemical oxygen demand, COD=chemical oxygen demand, FC=faecal coliforms, EC= E. coli, TC=total coliforms, FS=faecal streptococcus, TTC=thermos tolerant coliform. Microbiological units as cfc/100 mL unless stated otherwise, BDL=below detection limit. Notation: [1]Case-studies presenting data from a limited number of sites (n<20), limited temporal resolution as a single survey or use only basic chemical indicators and limited analysis of the results; [2] Case studies which either draw from larger data sets or include both chemical and microbiological indicators but have limited data analysis regarding sanitary risk factors; [3] Case studies with greater temporal resolution or are accompanied by a more thorough analysis of the data, for example using statistical techniques to understand the significance of different risk factors on water quality observations.

Table S2. Groundwater nitrate summary statistics for urban studies in SSA

Country	Town or city	Geology	source	Subset	Sample number	Sampling time-frame	NO3 MIN	NO3 MAX	NO3 MEAN	NO3 SD	Reference
Benin	Cotonou	Quaternary to mid Pleistocene sandstone	Wells		30	May 1991, August 1991 and April 1992	3.25	132	51.4	45.4	Boukari et al. (1996)
Cameroon	Douala	Alluvium over Pliocene sand and gravel	Springs and Boreholes		72	Single survey	0.21	94.3	32.5	28.7	Takem et al. (2010)
DR Congo	Kinshasa	Alluvial and sedimentary sequences	Wells	We and dry seasons	4	Seasonal study	76	198	119	39.8	Vala et al. (2011)
DR Congo	Brazaville	Alluvial and sedimentary sequences	Wells	We and dry seasons	23	Seasonal study	23	NA	1.32	0.64	Matini et al. (2012)
Ethiopia	Addis Ababa	Volcanics	Boreholes an d springs		9	Various	0.72	35	13	10	Abiye (2008)
Ethiopia	Mekelle	Mesozoic sediments	Wells, springs and boreholes		100	N/A	0.21	336	32.2	49.4	Berhane and Walraevens (2013)
Ghana	Kumasi	Precambrian Basement	Wells		10	N/A	0	0.968	0.25	0.33	Nkansah et al. (2010)
Guinea	Conakry	Volcanic rocks, fissured	Boreholes	Fountains	69	April-May 1994	0.00442	11.05	1.105	0.00442	Gélinas et al. (1996)
Guinea	Conakry	Volcanic rocks, fissured	Wells	Mod well	69	April-May 1994	52.156	203.32	151.606	52.156	Gélinas et al. (1996)
Guinea	Conakry	Volcanic rocks, fissured	Wells	Trad wells	69	April-May 1994	49.946	228.956	136.578	51.714	Gélinas et al. (1996)
Guinea Bissau	Bolama City	Sandy soils and Cenozoic-Modern sediments	Boreholes		28	Jul-06	0.9	55.3	16.6	2.4	Bordalo and Savva-Bordalo (2007)
Madacascar	Tamatave and Foulpointe	Weathered basement and unconsolidated sediments	Boreholes		53	Single study	4.4	35	23	12	MacCarthy et al. (2013)
Nigeria	Abeokuta	Basement igneous and metamorphic	Wells		25	Rainy season, 2008	2.97	40.7	17.6	10.6	Taiwo et al. (2011)

Nigeria	Calabar	Tertiary to recent sands and gravels	Wells		20	N/A	3.96	15.4	10	2.8	Eni et al. (2011)
Nigeria	Ilesha	Basement	Wells		87	Single survey	0.05	550	46.3	74.7	Malomo et al. (1990)
Nigeria	Lagos	Alluvium over sedimentary	Wells		18	Aug-Oct 2004	0.221	6.6742	1.768	1.8564	Yusuf (2007)
Nigeria	Masaka	Cretaceous sandstone and clay	Wells		12	Wet season but not during rainfall	44.5	92.5	61.5	20.3	Alhassan and Ujoh (2011)
Nigeria	Niamey	Basement	Wells		20	Monthly between July 1989 and April 1990	6.2	719	127.1	185.3	(Girard and Hillaire-Marcel, 1997)
Senegal	Dakar	Quaternary	Wells		56	July-October 1997	0	540	310	150	Cissé Faye et al. (2004)
Senegal	Dakar	Quaternary	Wells		47	July and November 1989	43	367.5	190	79.2	Tandia et al. (1999)
Sierra Leone	Kulanda	Weathered Granitic Basement	Wells	Wet season	33	Single study	2.21	123.7	34.2	33.1	Jimmy et al. (2013)
Southern Malawi		Weathered Basement	Wells		26	Wet and dry season	0.02	4.5	0.8	1	Pritchard et al. (2008)
Tanzania	Dodoma	Basement	Wells, springs and boreholes		49	Single survey	0.01	449	83.85	99.2	Nkotagu (1996)
Uganda	Kampala	Basement	Boreholes	Downgradient of Latrine	10	March-August 2010 biweekly sampling			228	237	Nyenje et al. (2013)
Uganda	Kampala	Basement	Springs		4	Wet and dry season for 5 consecutive weeks	4.3	50	17.2	12.4	Nsubuga et al. (2004)

Zambia	Lusaka	Dolomite	Wells		9	Jul-01	0.05	43	16.6	16.8	Cidu et al. (2003)
Zambia	Lusaka	Dolomite	Boreholes		14	Various: 1995-2000	0.03	110	26.6	31.4	Nkhuwa (2003)
Zimbabwe	Bulawayo	Granite and Greenstone	Boreholes	We and dry seasons	32	Seasonal study	0.05	44	4.2	5.3	Mangore and Taigbenu (2004)
Zimbabwe	Epworth, Harare	Granite	Wells and boreholes		18	Survey carried out with duplicate sampling	0	30	11.46	10.36	Zingoni et al. (2005)

Table S3. Factors affecting transport and attenuation of microorganisms in groundwater (from Pedley et al. (2006))

Characteristics of the microorganism	Aquifer/soil (environment) properties
Size	Groundwater flow velocity
Shape	Dispersion
Density	Pore size (intergranular or fracture)
Inactivation rate (die-off)	Kinematic/effective porosity
(Ir)reversible adsorption	Organic carbon content and nature of OC
Physical filtration	Temperature
	Chemical properties of groundwater (nutrients, redox, pH etc.)
	Mineral composition of aquifer/soil material
	Predatory microflora
	Moisture content
	Pressure

References for Tables in Supporting Information

ABIYE, T A. 2008. Urban groundwater pollution in Addis Ababa, Ethiopia. 261-276 in Applied groundwater studies in Africa. ADELANA, S M A, and MACDONALD, A M (editors). 13. (CRC.)

ADELEKAN, B A, and ALAWODE, A O. 2011. Contributions of municipal refuse dumps to heavy metals concentrations in soil profile and groundwater in Ibadan Nigeria Journal of Applied Biosciences, Vol. 40, 2727-2737.

ALEMAYEHU, T. 2006. Heavy metal concentration in the urban environment of Addis Ababa, Ethiopia. Soil and Sediment Contamination: An International Journal, Vol. 15, 591-602.

Aller L, Bennet T, Lehr J.H, Petty R, 1987. National Water Well Association. DRASTIC: A standardized system for evaluating groundwater pollution potential using hydrogeologic settings. EPA/600/2-85/018, U.S. Environ. Prot. Agency, Ada, Oklahoma.

ALHASSAN, M M, and UJOH, F. 2011. An assessment of ground water quality for drinking from hand-dug wells in Masaka, Nigeria. Bayero University Journal of Social and Management Studies, Vol. 14, 79-95.

AREMU, D A, OLAWUYI, J F, MESHITSUKA, S, SRIDHAR, M K, and OLUWANDE, P A. 2002. Heavy metal analysis of groundwater from Warri, Nigeria. International Journal of Environmental Health Research, Vol. 12, 261-267.

BARRETT, M H, HOWARD, A G, PEDLEY, S, TAYLOR, R G, and NALUBEGA, M. 1998. A comparison of the extent and impacts of sewage contamination on urban groundwater in developed and developing countries. WHO conference: Water, Sanitation and Health, 24-28 November, Bad Elster, Germany.

BERHANE, G, and WALRAEVENS, K. 2013. Geological and geotechnical constraints for urban planning and natural environment protection: a case study from Mekelle City, Northern Ethiopia. Environmental Earth Sciences, Vol. 69, 783-798.

BORDALO, A A, and SAVVA-BORDALO, J. 2007. The quest for safe drinking water: An example from Guinea-Bissau (West Africa). Water Research, Vol. 41, 2978-2986.

BOUKARI, M, GAYE, C B, FAYE, A, and FAYE, S. 1996. The impact of urban development on coastal aquifers near Cotonou, Benin. Journal of African Earth Sciences, Vol. 22, 403-408.

BRAUNE, E, and XU, Y. 2010. The role of ground water in Sub-Saharan Africa. Ground Water, Vol. 48, 229-238.

CIDU, R, DE WAELE, J, DI GREGARIO, F, and FOLLESA, R. 2003. Geochemistry of groundwater in an intensely urbanised karst area (Lusaka, Zambia) GeoActa, Vol. 2, 35-42.

CISSÉ FAYE, S, FAYE, S, WOHNLICH, S, and GAYE, C. 2004. An assessment of the risk associated with urban development in the Thiaroye area (Senegal). Environmental Geology, Vol. 45, 312-322.

CRONIN, A A, PEDLAY, S, HOADLY, A W, KOUONTO KOMOU, F, HALDIN, L, GIBSON, J, and BRESLIN, N. 2007. Urbanisation effects on groundwater chemical quality: findings focusing on the nitrate problem from 2 African cities reliant on on-site sanitation. Journal of Water and Health, Vol. 5, 441-454.

DEMLIE, M, and WOHNLICH, S. 2006. Soil and groundwater pollution of an urban catchment by trace metals: case study of the Addis Ababa region, central Ethiopia. Environmental Geology, Vol. 51, 421-431.

ENI, D, OBIEFUNA, J N, OKO, C, and EKWOK, I. 2011. Impact of urbanisation on sub-surface water quality in Calabar Municipality, Nigeria. International Journal of Humanities and Social Science, Vol. 1, 167-172.

ERAH, P O, and AKUJIEZE, C N. 2002. The quality of groundwater in Benin City: A baseline study on inorganic chemicals and microbial contaminants of health importance in boreholes and open wells. Tropical Journal of Pharmaceutical Research, Vol. 1, 75-82.

EWODO, M G, EKWELGEN, C, NTEP, F, and EKODECK, G E. 2009. Impact of urbanisation on the Mingosso watershed in the Yaounde periurban zone. African Journal of Environmental Science and Technology, Vol. 3, 272-285.

FAILLAT, J P. 1990. Sources of Nitrates in Fissure Groundwater in the Humid Tropical Zone - the Example of Ivory-Coast. Journal of Hydrology, Vol. 113, 231-264.

GÉLINAS, Y, RANDALL, H, ROBIDOUX, L, and SCHMIT, J-P. 1996. Well water survey in two districts of Conakry (Republic of Guinea), and comparison with the piped city water. Water Research, Vol. 30, 2017-2026.

GIRARD, P, and HILLAIRE-MARCEL, C. 1997. Determining the source of nitrate pollution in the Niger discontinuous aquifers using the natural 15N/14N ratios. Journal of Hydrology, Vol. 199, 239-251.

GOSHU, G, and AKOMA, O C. 2011. Water quality assessment of underground and surface water resources of Bahir Dar and Periurban areas, north-west Ethiopia. Global Journal of Environmental Sciences, Vol. 10, 11-21.

GOSHU, G, FARNLEITNER, A, MANAFI, M, and BYAMUKAMA, D. 2010. The bacteriological quality of traditional hand dug wells and protected hand pumps in Bahirdar Town and peri-urban areas, Northern Ethiopia. Proceedings of the First National Research Symposium on: Sustainable Development: A great concern in Africa, Debre Markos, Ethiopia, 247-259.

HOWARD, G, PEDLEY, S, BARRETT, M, NALUBEGA, M, and JOHAL, K. 2003. Risk factors contributing to microbiological contamination of shallow groundwater in Kampala, Uganda. Water Research, Vol. 37, 3421-3429.

IBE, K M, and AGBAMU, P U. 1999. Impacts of human activities on groundwater quality of an alluvial aquifer: A case study of the Warri River, Delta State, SW, Nigeria. International Journal of Environmental Health Research, Vol. 9, 329-334.

JIMMY, D H, SUNDUFU, A J, MALANOSKI, A P, JACOBSEN, K H, ANSUMANA, R, LESKI, T A, BANGURA U, BOCKARIE A S, TEJAN E, LIN, B, and STENGER, D A. 2013. Water quality associated public health risk in Bo, Sierra Leone. Environmental monitoring and assessment, 185, 1, 241-251.

KIMANI-MURAGE, E, and NGINDU, A. 2007. Quality of water the slum dwellers use: The case of a Kenyan slum. Journal of Urban Health, Vol. 84, 829-838.

Liddle, E.S., Mager, S.M. and Nel, E.L., 2014. The importance of community-based informal water supply systems in the developing world and the need for formal sector support. *The Geographical Journal*.

MACCARTHY, M F, ANNIS, J E, and MIHELCIC, J R. 2013. Unsubsidised Self-Supply in Eastern Madagascar. Water Alternatives, Vol. 6, 424-438.

Mailu, G. M. (1997). The impact of urbanization on groundwater quality in Wajir Town, Kenya. IAHS Publication, 243, 245-253.

MALOMO, S, OKUFARASIN, V A, OLORUNNIWO, M A, and OMODE, A A. 1990. Groundwater Chemistry of Weathered Zone Aquifers of an Area Underlain by Basement-Complex Rocks. Journal of African Earth Sciences, Vol. 11, 357-371.

Matini, L., Tathy, C. and Moutou, J. M. 2012. Seasonal Groundwater Quality Variation in Brazzaville, Congo. Research Journal of Chemical Sciences 2(1): 7-14.

MOMODU, M A, and ANYAKORA, C A. 2010. Heavy metal contamination of ground water: The Surulere case study. Research Journal Environmental and Earth Sciences, Vol. 2, 39-43.

NACHIYUNDE, K, IKEDA, H, TANAKA, K, and KOZAKI, D. 2013. Evaluation of portable water in five provinces of Zambia using a water pollution index. African Journal of Environmental Science and Technology, Vol. 7, 14-29.

NKANSAH, M A, BOADI, N O, and BADU, M. 2010. Assessment of the quality of water from hand-dug wells in Ghana. Environmental Health Insights., Vol. 4, 7-12.

NKHUWA, D C W. 2006. Groundwater quality assessment in the John Laing and Misisi areas of Lusaka. 239-252 in Groundwater pollution in Africa. XU, Y, and USHER, B (editors). (Leiden: Taylor & Francis/Balkema.)

NKOTAGU, H. 1996. Origins of high nitrate in groundwater in Tanzania. Journal of African Earth Sciences, Vol. 22, 471-478.

NSUBUGA, F B, KANSIIME, F, and OKOT-OKUMU, J. 2004. Pollution of protected springs in relation to high and low density settlements in Kampala—Uganda. Physics and Chemistry of the Earth, Parts A/B/C, Vol. 29, 1153-1159.

NYENJE, PM, FOPPEN, JW, KULABAKO, R, MUWANGA, A, AND UHLENBROOK, S. 2013. Nutrient pollution in shallow aquifers underlying pit latrines and domestic solid waste dumps in urban slums. Journal of environmental management, 122, 15-24.

OBIRI-DANSO, K, ADJEI, B, STANLEY, K, and JONES, K. 2009. Microbiological quality and metal levels in wells and boreholes water in some peri-urban communities in Kumasi, Ghana. African Journal of Environmental Science & Technology, Vol. 3, 59-66.

OCHIENG, G M, OJO, O I, OGEDENGBE, K, and NDAMBUKI, J M. 2011. Open wells, sanitary features, pollutions and water qualities: case study of Ibadan slums, Nigeria International Journal of the Physical Sciences, Vol. 6, 3062-3073.

OLABISI, O E, AWONUSI, A J, and ADEBAYO, O J. 2008. Assessment of bacteria pollution of shallow well water in Abeokuta, Southwestern Nigeria. Life Science Journal, Vol. 5, 68-72.

OREBIYI, E O, AWOMESO, J A, IDOWU, O A, MARTINS, O, OGUNTOKE, O, and TAIWO, A M. 2010. Assessment of pollution hazards of shallow well water in Abeokuta and environs, Southwest, Nigeria. American Journal of Environmental Sciences, Vol. 6, 50-56.

Ouandaogo-Yameogo, S., Blavoux, B., Nikiema, J. and Savadogo, A.N., 2013. Caractérisation du fonctionnement des aquifères de socle dans la région de Ouagadougou à partir d'une étude de la qualité chimique des eaux.*Revue des sciences de l'eau/Journal of Water Science*, *26*(3), pp.173-191.

PALAMULENI, L G. 2002. Effect of sanitation facilities, domestic solid waste disposal and hygiene practices on water quality in Malawi's urban poor areas: a case study of South Lunzu Township in the city of Blantyre. Physics and Chemistry of the Earth, Parts A/B/C, Vol. 27, 845-850.

PEDLEY, S, YATES, M, SCHIJVEN, J F, WEST, J, HOWARD, G, BARRETT, M, SCHMOLL, O, CHILTON, J, and CHORUS, I. 2006. Pathogens: health relevance, transport and attenuation. Protecting groundwater for health: managing the quality of drinking-water sources. (Geneva: WHO.) ISBN 92-4-154668-9

PRITCHARD, M, MKANDAWIRE, T, and O'NEILL, J G. 2008. Assessment of groundwater quality in shallow wells within the southern districts of Malawi. Physics and Chemistry of the Earth, Parts A/B/C, Vol. 33, 812-823.

Sorensen, J.P.R., Lapworth, D.J., Marchant, B.P., Nkhuwa, D.C.W., Pedley, S., Stuart, M.E., Bell, R.A., Chirwa, M., Kabika, J., Liemisa, M. and Chibesa, M., 2015b. In-situ tryptophan-like fluorescence: a real-time indicator of faecal contamination in drinking water supplies. Water research, 81, pp.38-46.

Sorensen, J.P.R., Lapworth, D.J., Nkhuwa, D.C.W., Stuart, M.E., Gooddy, D.C., Bell, R.A., Chirwa, M., Kabika, J., Liemisa, M., Chibesa, M. and Pedley, S., 2015c. Emerging contaminants in urban groundwater sources in Africa. Water research, 72, pp.51-63.

Tabor, M., Kibret, M. and Abera, B., 2011. Bacteriological and physicochemical quality of drinking water and hygiene-sanitation practices of the consumers in Bahir Dar City, Ethiopia. *Ethiopian journal of health sciences*, *21*(1), pp.19-26.

TAIWO, A M, ADEOGUN, A O, OLATUNDE, K A, and ADEGBITE, K I. 2011. Analysis of groundwater quality of hand-dug wells in peri-urban area of Obantoko, Abeokuta, Nigeria for selected physico-chemical parameters. The Pacific Journal of Science and Technology, Vol. 12, 527-534.

TAKEM, G E, CHANDRASEKHARAM, D, AYONGHE, S N, and THAMBIDURAI, P. 2010. Pollution characteristics of alluvial groundwater from springs and bore wells in semi-urban informal settlements of Douala, Cameroon, Western Africa. Environmental Earth Sciences, Vol. 61, 287-298.

VALA, R M K, TICHAGWA, L, MUSIBONO, D E, and LUKANDA, V M. 2011. Environmental and health concerns regarding the quality of water in a poor suburb of Kinshasa in the Democratic Republic of Congo. Water Science & Technology: Water Supply, Vol. 11, 266-273.

Wright, J.A., Cronin, A., Okotto-Okotto, J., Yang, H., Pedley, S. and Gundry, S.W., 2013. A spatial analysis of pit latrine density and groundwater source contamination. Environmental monitoring and assessment,185 (5), pp.4261-4272.

YUSUF, K A. 2007. Evaluation of groundwater quality characteristics in Lagos City. Journal of Applied Sciences, Vol. 7, 17980-11784.

ZINGONI, E, LOVE, D, MAGADZA, C, MOYCE, W, and MUSIWA, K. 2005. Effects of a semi-formal urban settlement on groundwater quality: Epworth (Zimbabwe): Case study and groundwater quality zoning. Physics and Chemistry of the Earth, Parts A/B/C, Vol. 30, 680-688.
